# NAD^+^ augmentation restores mitophagy and limits accelerated aging in Werner syndrome

**DOI:** 10.1038/s41467-019-13172-8

**Published:** 2019-11-21

**Authors:** Evandro F. Fang, Yujun Hou, Sofie Lautrup, Martin Borch Jensen, Beimeng Yang, Tanima SenGupta, Domenica Caponio, Rojyar Khezri, Tyler G. Demarest, Yahyah Aman, David Figueroa, Marya Morevati, Ho-Joon Lee, Hisaya Kato, Henok Kassahun, Jong-Hyuk Lee, Deborah Filippelli, Mustafa Nazir Okur, Aswin Mangerich, Deborah L. Croteau, Yoshiro Maezawa, Costas A. Lyssiotis, Jun Tao, Koutaro Yokote, Tor Erik Rusten, Mark P. Mattson, Heinrich Jasper, Hilde Nilsen, Vilhelm A. Bohr

**Affiliations:** 1 Laboratory of Molecular Gerontology, National Institute on Aging, National Institutes of Health, Baltimore, MD 21224 USA; 2 Department of Clinical Molecular Biology, University of Oslo and Akershus University Hospital, 1478 Lørenskog, Norway; 3 Buck Institute for Research on Aging, Novato, CA 94945 USA; 4 Department of Molecular Cell Biology, Institute for Cancer Research, Oslo University Hospital, Montebello, N-0379 Oslo Norway; 5 Centre for Cancer Biomedicine, Faculty of Medicine, University of Oslo, Montebello, N-0379 Oslo Norway; 6 Laboratory of Neurosciences, National Institute on Aging, National Institutes of Health, Baltimore, MD 21224 USA; 7 Danish Center for Healthy Aging, University of Copenhagen, Blegdamsvej 3B, 2200 Copenhagen, Denmark; 8 Department of Molecular and Integrative Physiology, University of Michigan Medical School, Ann Arbor, MI 48109 USA; 9 Clinical Cell Biology and Medicine, Chiba University Graduate School of Medicine, 1–8–1 Inohana, Chuo-ku, Chiba 260–8670 Japan; 10 Molecular Toxicology Group, Department of Biology, University of Konstanz, 78457 Konstanz, Germany; 11 Department of Molecular and Integrative Physiology, and Department of Internal Medicine, University of Michigan Medical School, Ann Arbor, MI 48109 USA; 12 Department of Hypertension and Vascular Disease, the First Affiliated Hospital, Sun Yat-Sen University, 510080 Guangzhou, China; 13 Department of Neuroscience, Johns Hopkins University School of Medicine, Baltimore, MD 21205 USA

**Keywords:** Mitophagy, Diabetes complications

## Abstract

Metabolic dysfunction is a primary feature of Werner syndrome (WS), a human premature aging disease caused by mutations in the gene encoding the Werner (WRN) DNA helicase. WS patients exhibit severe metabolic phenotypes, but the underlying mechanisms are not understood, and whether the metabolic deficit can be targeted for therapeutic intervention has not been determined. Here we report impaired mitophagy and depletion of NAD^+^, a fundamental ubiquitous molecule, in WS patient samples and WS invertebrate models. WRN regulates transcription of a key NAD^+^ biosynthetic enzyme nicotinamide nucleotide adenylyltransferase 1 (NMNAT1). NAD^+^ repletion restores NAD^+^ metabolic profiles and improves mitochondrial quality through DCT-1 and ULK-1-dependent mitophagy. At the organismal level, NAD^+^ repletion remarkably extends lifespan and delays accelerated aging, including stem cell dysfunction, in *Caenorhabditis elegans* and *Drosophila melanogaster* models of WS. Our findings suggest that accelerated aging in WS is mediated by impaired mitochondrial function and mitophagy, and that bolstering cellular NAD^+^ levels counteracts WS phenotypes.

## Abstract (web summary)

The molecular mechanisms of mitochondrial dysfunction in the premature ageing Werner syndrome were elusive. Here the authors show that NAD^+^ depletion-induced impaired mitophagy contributes to this phenomenon, shedding light on potential therapeutics.

## Introduction

Werner syndrome (WS) is an autosomal recessive accelerated aging disorder with major clinical phenotypes including cancer, short stature, graying and loss of hair, prematurely aged faces, juvenile cataracts, dyslipidemia, premature atherosclerosis, and insulin-resistant diabetes^1–3^. The incidence of fatty liver is 42% in WS patients, 60% of them have dyslipidemia, and 70% have diabetes^1^. With treatments for associated diseases and for removal of symptoms, the life quality for these individuals could be improved. However, there is no current therapy for WS. Some features of WS may be explained by genomic instability due to mutation in the gene encoding the Werner protein (WRN), an important DNA helicase/exonuclease involved in DNA repair (e.g., double-strand break repair/DSBR, base excision repair/BER), telomere and heterochromatin maintenance, and cancer regulation^4–7^. However, the relationship between *WRN* mutations and the syndrome’s severe dysregulation of energy metabolism is unclear^1^.

Mitochondrial quality and function decline with age, contributing to insulin resistance and metabolic diseases in the elderly^8–11^. Mitochondrial quality control is regulated by biogenesis and mitophagy^10,12^. Mitophagy involves the targeting of damaged mitochondria to the lysosomes wherein the mitochondrial constituents are degraded and recycled^13^. Defective mitophagy is prominent in aging and age-predisposed disorders, including metabolic diseases and neurodegeneration^12,14^. However, the role of mitophagy in WS has not been investigated. The metabolic molecule nicotinamide adenine dinucleotide (NAD^+^) is emerging as a fundamental regulator of mitochondrial homeostasis, genome stability, neuroprotection, healthy aging and longevity^12,15–17^. Interestingly, genetic and/or pharmacological upregulation of intracellular NAD^+^ levels protects against obesity and type 2 diabetes in rodents^14,18–20^, and against age-related diseases and neurodegenerative diseases such as Alzheimer’s disease^16,17,21–25^.

We therefore examined whether mitochondrial dysfunction and NAD^+^ depletion occur in WS, and if so, how it contributes to the molecular pathology in WS. We report that NAD^+^ depletion is a major driver of the severe metabolic dysfunction in WS through dysregulation of mitochondrial homeostasis. NAD^+^ augmentation extends lifespan and healthspan in both *C. elegans* and *Drosophila* models of WS. Understanding how WRN affects metabolism has important implications for elucidating the mechanism of accelerated aging in WS and for therapeutic strategies for this currently incurable disease and possibly other age-related diseases.

## Results

### Mitochondrial alterations and NAD^+^ depletion in WS

**Fig. 1:**
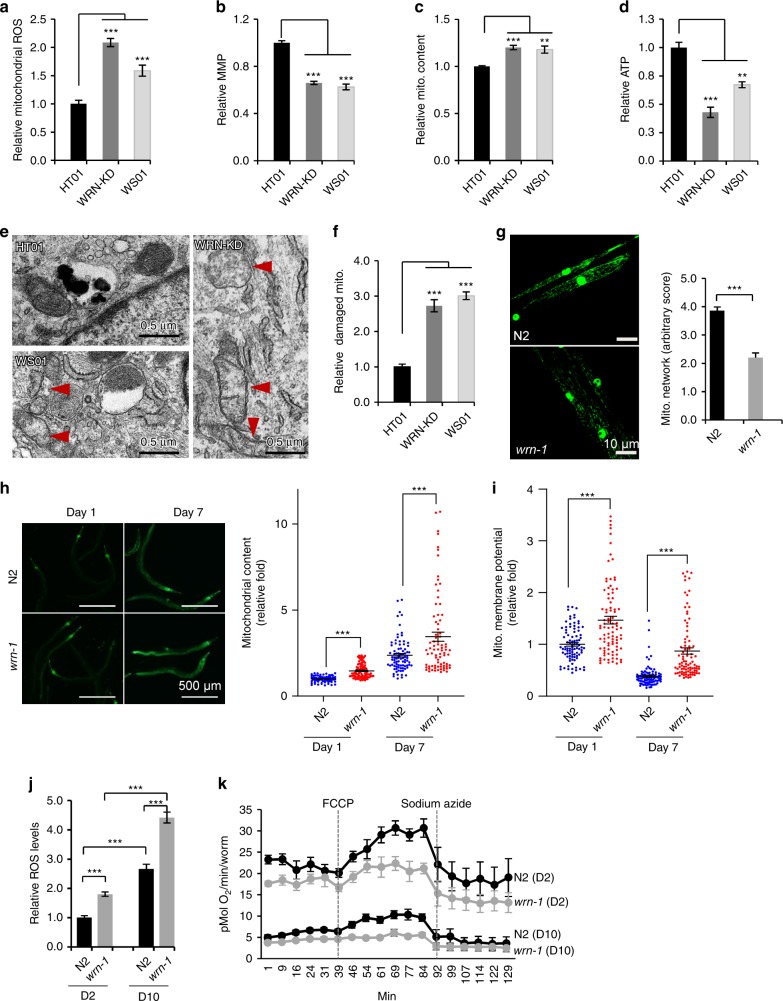
Mitochondrial dysfunction and NAD^+^ reduction in WS human cells and *C. elegans*. (**a**–**c**) Flow cytometry was used to quantify relative mitochondrial ROS (**a**, mitoSOX), mitochondrial membrane potential (**b**, TMRM), and mitochondrial content (**c**, MitoTracker Green). *n* = 3 biologically independent experiments (One-way ANOVA). **d** Cellular ATP levels. *n* = 3 biologically independent experiments (One-way ANOVA). **e**, **f** Changes of mitochondrial ultrastructure were evaluated through electron microscopy (**e**) and damaged mitochondria quantified (**f**). (*n* = 100 mitochondria from three independent samples; One-way ANOVA). Red arrows denote damaged mitochondria. **g** A *myo-3::gfp* reporter was expressed in both nucleus and mitochondria to mark non-pharyngeal body wall muscle cells in worms. Representative images and quantified scores of muscle mitochondrial morphology of adult D2 N2 and *wrn-1*(*gk99*) worms. Data are shown in mean ± S.E.M (*n* = 20 worms; Student *t*-test). **h** Increased organismal mitochondrial content in the WS worms. MitoTracker Green was used for organismal mitochondrial staining. A set of representative images (Scale bar, 500 µm) is shown with quantified data in mean ± S.E.M. with values pooled from three independent biological repeats (*n* = 80 worms; Two-way ANOVA). **i** Upregulated mitochondrial membrane potential (MMP) in the WS worms. Data are shown in mean ± S.E.M. with values pooled from three independent biological repeats (*n* = 90 worms; Two-way ANOVA). **j** Relative levels of ROS in adult D2 and D10 worms. (*n* = 10 worms/group; Two-way ANOVA). **k** OCR in adult D2 and D10 worms (*n* = 15 worms/well, three biological repeats with quantified data). Data are shown in mean ± S.E.M. *p *<* 0.05, **p *<* 0.01, ***p *<* 0.001.

We speculated that the abnormal glucose and lipid metabolism in WS^1^ could be caused by mitochondrial dysfunction and evaluated a series of mitochondrial parameters using primary fibroblasts from a 30-year WS patient (termed WS01). These were compared to primary fibroblasts from a sex- and age-matched healthy control subject (termed HT01) (Supplementary Table 1). We also created an isogenic cell line by using siRNA to deplete *WRN* in normal control fibroblasts (termed WRN-KD). WS01 and WRN-KD cells had higher mitochondrial ROS, lower mitochondrial membrane potential, increased mitochondrial content, and decreased cellular ATP levels compared to HT01 cells (Fig. 1a-d). To explore the underlying causes of abnormal mitochondria in WS, we evaluated mitochondrial ultrastructure using electron microscopy (EM). WRN deficient cells exhibited a nearly 3-fold increase in damaged mitochondria relative to HT01 cells, including loss of cristae morphology and reduced density (Fig. 1e, f). We then asked whether mitochondrial dysfunction is conserved across animal models of WS. We started with a *C. elegans* model of WS, *wrn-1(gk99)*, which has a 196-bp deletion mutation resulting in the complete absence of the WRN-1 protein^26,27^. This strain recapitulates major features of WS patients, including impaired DSBR, progeroid tissue phenotypes, and shorter lifespan^3,27,28^. Similar to the observations in human WS cells, we found reduced mitochondrial network complexity (42% reduction, Fig. 1g) using MYO-3::GFPincreased organismal mitochondrial content (Fig. 1h), increased mitochondrial membrane potential (Fig. 1i), and increased oxidative stress (Fig. 1j) in *wrn-1(gk99)* compared to wild type (WT) N2 worms., Both young (adult Day 2/D2) and old (D10) *wrn-1(gk99)* worms exhibited decreased basal and maximal mitochondrial oxygen consumption (OCR) rates compared to N2 worms (Fig. 1k, quantification and statistics in supplementary Fig. 1). The mitochondrial phenotypes in WS human cells and animals are similar to the phenotypes we reported in other premature ageing diseases^21,29^. There was no detectable mitochondrial impairment in mouse embryonic fibroblasts (MEFs), brain, liver, or heart tissue from *Wrn*^−*/−*^ mice compared with samples from matched WT littermates (Supplementary Fig. 2a–e). It is well established that WS mice do not recapitulate the human phenotype^30^. This may relate to its long telomeres, which dilute the importance of the DNA repair/telomere maintaining functions.

**Fig. 2:**
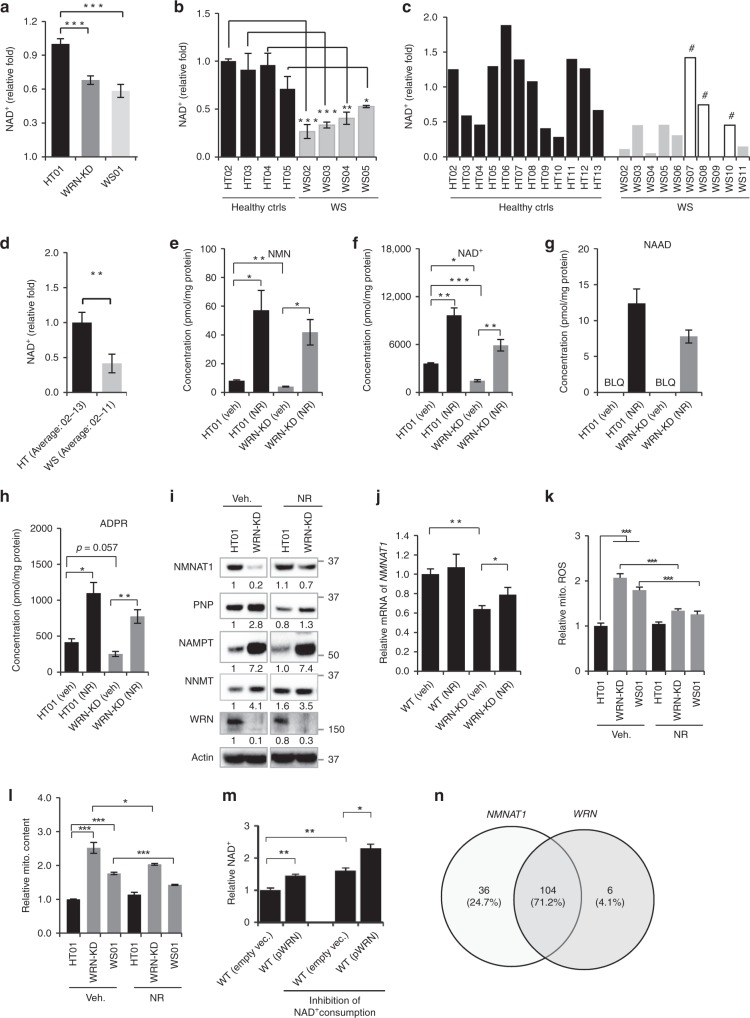
Impaired NAD^+^-generating machinery in human WS cells. **a**, **b** Relative NAD^+^ levels in human WS patient cells and controls. Data, mean ± S.E.M (*n* = 3 biologically independent experiments; One-way ANOVA). **c**, **d** Relative NAD^+^ levels in blood samples from human WS patients and controls. Data of **d** were mean ± S.E.M from all samples of **c**. #, samples from WS patients without obesity (also see Supplementary Table 2). One-way ANOVA or Student *t*-test was used for data analysis (**e**–**h**) LC-MS data showing changes of NMN (**e**), NAD^+^ (**f**), NAAD (**g**), and ADPR (**h**) in HT01 and WRN-KD cells before and after NR treatment (1 mM, 24 h). (*n* = 3 biologically independent experiments) (One-way ANOVA). **i** WRN regulates NMNAT1 at protein level. Source data are provided as a Source Data file. **j** WRN regulates NMNAT1 at transcriptional level. mRNA levels of *NMNAT1* in different conditions were measured using real-time PCR. Data are shown in mean ± S.E.M (*n* = 3 biologically independent experiments; One-way ANOVA). **k**, **l** Effect of NR (1 mM, 24 h) on the relative levels of mitochondrial ROS (mitoSOX dye) and mitochondrial content (mitoGreen dye) of the designated cells. Data are shown in mean ± S.E.M (*n* = 3 biologically independent experiments; One-way ANOVA). **m** Relative NAD^+^ levels in WT or *WRN*-overexpressing cells. Data are shown in mean ± S.E.M (*n* = 3 biologically independent experiments; One-way ANOVA). **n** Venn diagram with transcription factors that bind the genes respective promoters of *NMNAT1* and *WRN* in ChIP-seq datasets from the ENCODE Transcription factor target datasets. Data are shown in mean ± S.E.M. *p *<* 0.05, **p *<* 0.01, ***p *<* 0.001.

Alterations in mitochondrial function and concurrent NAD^+^ reduction are common in aging and metabolic disorders^16,31^. NAD^+^ is a central cellular metabolite in health and aging modulating the activity of proteins involved in genomic maintenance, cellular bioenergetics, and adaptive stress responses^15,16^. NAD^+^ levels in WS01 or WRN-KD cells were 30–40% lower than in HT01 cells (Fig. 2a). We also examined NAD^+^ levels in other primary fibroblasts and in plasma from WS patients and healthy controls (Supplementary Tables 1 and 2). NAD^+^ levels in four different WS cell lines (WS2-WS5) ranged from 20 to 70% of matched healthy controls (HT2-HS5, Fig. 2b). We further examined NAD^+^ in plasma from 10 WS patients (WS02-WS11) and 12 healthy controls (HT2-HT13). Although NAD^+^ levels varied within each group, the average NAD^+^ was 58% lower in WS than healthy control fibroblasts (Fig. 2c, d). Notably, the three WS plasma samples with the highest NAD^+^ levels were from patients that did not exhibit diabetic phenotypes (Fig. 2c marked with # and Supplementary Table 2), suggesting a possible negative correlation between NAD^+^ and the severity of metabolic dysfunction in WS patients. Verification of NAD^+^ reduction in whole blood samples from larger WS patient samples are necessary. In summary, these results indicate NAD^+^ depletion across species in WS.

### NAD^+^ replenishment normalizes NAD^+^ biosynthesis

After observing a reduction of NAD^+^ in WS patient cells and plasma, and in a *C. elegans* model of WS, we asked whether this alteration drove the accelerated aging phenotypes in WS. We increased cellular NAD^+^ levels by incubating the HT01 and WRN-KD cells with the NAD^+^ precursor nicotinamide riboside (NR, 1 mM for 24 h), followed by systematic evaluation of intra- and extracellular NAD^+^ metabolic profiles using liquid chromatography-mass spectrometry (LC-MS)^32,33^. NAD^+^ and its precursor nicotinamide mononucleotide (NMN) were both decreased in WRN-KD cells relative to in HT01 cells (Fig. 2e, f). NR treatment robustly increased NMN and NAD^+^ levels in both WRN-KD and HT01 cells (Fig. 2e, f). Nicotinic acid adenine dinucleotide (NAAD) is the substrate of glutamine-dependent NAD^+^ synthetase^15^. NAAD levels were undetectable in fibroblasts not treated with NR and increased to ~7 and 12 pmol/mg protein in NR-treated WRN-KD and HT01 cells, respectively (Fig. 2g). This is in line with a recent clinical study of NR in healthy humans, suggesting that NAAD is a highly sensitive biomarker for effective NAD^+^ supplementation^32^. NR treatment also led to increased ADPR (Fig. 2h). There was a significant decrease in methylated nicotinamide (MeNAM) in WRN-KD cells relative to WT, however, no significant change in MeNAM after NR treatment was observed (Supplementary Fig. 3a). There were no clear differences in other NAD^+^ related metabolites, such as inosine, inosine 5′-monophosphate (IMP), and NADP in the NR-treated WRN-KD cells compared to WRN-KD (veh) cells (Supplementary Fig. 3b–i). We further examined extracellular NAD^+^ metabolic profiles using medium from cells that had been treated with NR for 24 h. The extracellular metabolomic data show that nicotinamide (NAM) and nicotinate levels were dramatically increased by ~3- to 20-fold after NR treatment relative to the medium from vehicle-treated control cells, indicating increased NAD^+^ metabolism in the cells although we can not exclude a possibility of partial NR degradation (Supplementary Fig. 4).

Cellular NAD^+^ is tightly regulated by a series of enzymes involved in NAD^+^ synthesis and consumption^15,16,31^. We examined the protein expression levels of these enzymes. Nicotinamide nucleotide adenylyltransferase 1 (NMNAT1) is a key NAD^+^ biosynthetic enzyme, which catalyzes the formation of NAD^+^ from NMN and is primarily located in the nucleus^15,16^. Compared with HT01 cells, the level of total NMNAT1 was decreased in WRN-KD primary cells (Fig. 2i and quantification in Supplementary Fig. 3j). NMNAT1 was also decreased in other cells with WRN KD, e.g., in the U2OS cells (Supplementary Fig. 3k). We asked whether WRN regulates NMNAT1 at the transcriptional level. We knocked down *WRN* in human U2OS cells, which resulted in a 40% decrease in *NMNAT1* (Fig. 2j). mRNA and protein levels of MMNAT1 increased in response to NR treatment (Fig. 2i, j, Supplementary Fig. 3j). Nicotinamide phosphoribosyltransferase (NAMPT) is the rate-limiting enzyme in the NAD^+^ salvage pathway, which recycles NAM to NMN for NAD^+^ biosynthesis^15,16^. Notably, the protein levels of NAMPT in the WRN-KD cells were higher than in the HT01 cells, suggesting a compensatory cellular feedback to increase NAD^+^ synthesis (Fig. 2i). There were no significant changes in levels of the NAD^+^-consuming CD38 or CD157^34^ in the WRN-KD cells (Supplementary Fig. 3l). The protein and activity levels (as shown by PARylation) of poly(ADP-ribose)polymerases (PARPs) in the WRN deficient cells were higher compared with HT01 (veh) cells (Supplementary Fig. 3m). There was also a trend towards increased PARylation in *wrn-1(gk99)* worms relative to N2 controls; however, there was no significant difference of PARylation in either the NR-treated *wrn-1(gk99)* or N2 worms (Supplementary Fig. 3n). NAD^+^ repletion reduced mitochondrial oxidative stress and mitochondrial content in the WRN-KD and WS01 cells (Fig. 2k, l). To understand the role of WRN in maintaining NAD^+^ levels, the HT01 cells were transfected with either an empty vector or a *WRN*-encoding vector. To inhibit concurrent cellular NAD^+^ consumption, a small compound cocktail containing inhibitors of sirtuins, CD38, and PARPs was used. WRN overexpression in human fibroblasts lead to an increase in NAD^+^ levels (Fig. 2m). WRN participates in transcription^35,36^, and we checked for potential co-regulation by evaluating known transcription factors that bind to the respective gene promoters in ChIP-seq datasets from the ENCODE Transcription factor target datasets and WRN data. The analysis suggests that *NMNAT1* has 140 targets while *WRN* has 110. Interestingly, *NMNAT1* and *WRN* share most of their known targets, including SIRT6, WRNIP, BRCA1, BACH1, and RAD21 (Fig. 2n). A summary of the changes in NAD^+^ metabolites and in related enzyme levels before and after NR treatment is shown in Supplementary Fig. 3o. Collectively, these results suggest that WRN depletion induces NAD^+^ depletion and an imbalance of the NAD^+^ synthetic machinery, while NR treatment corrects these defects.

### NAD^+^ replenishment inhibits accelerated aging in WS animals

**Fig. 3:**
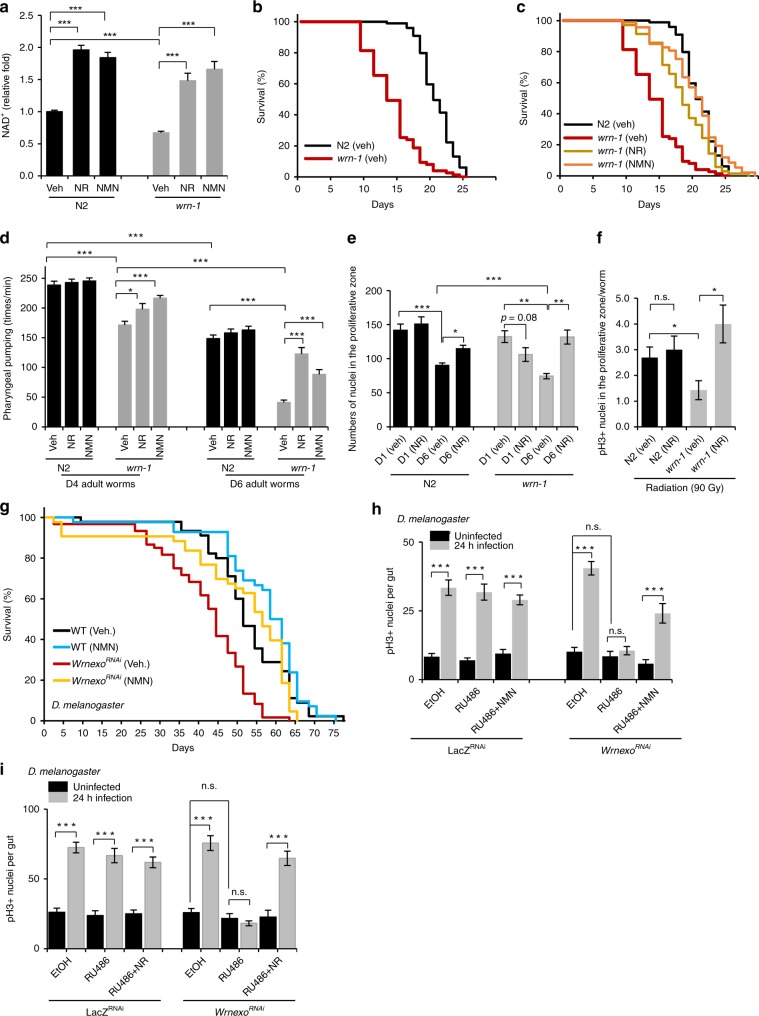
NAD^+^ replenishment ameliorates accelerated aging in WS worms and flies. **a** Relative NAD^+^ levels in adult D2 worms. Data are shown in mean ± S.E.M (*n* = 3 biologically independent experiments; Two-way ANOVA). **b** Lifespan curves in N2 and *wrn-1*(*gk99*) worms at 25 °C. **c** Effects of NR/NMN on lifespan in *wrn-1*(*gk99*) worms at 25 °C. Experiments were repeated seven times. One representative set of data is shown. Summarized data, including effects of NR/NMN on N2 worms, are in Supplementary Table 4. **d** Effects of NR/NMN on pharyngeal pumping in N2 and *wrn-1*(*gk99*) worms. Data, mean ± S.E.M (*n* = 3 biologically independent experiments with 10–20 worms for each condition; Two-way ANOVA). **e** Changes of germ line-localized mitotic cells in designated groups of worms. Data, mean ± S.E.M (*n* = 10 worms per condition; Two-way ANOVA). **f** Differences of the numbers of pH3^+^ mitotic cells in the designated groups of worms after exposure to 90 Gy ionizing radiation. Data, mean ± S.E.M (*n* = 6 worms per condition; Two-way ANOVA). **g** Effects of NAD^+^ supplementation on lifespan in *w;DaGeneSwitch-Gal4;y,v,sc;Wrnexo*^RNAi^ (EtOH) (WT), and *w;DaGeneSwitch-Gal4;y,v,sc;Wrnexo*^RNAi^ (RU486) (WRN-KD) flies at 25 °C without/with 5 mM NMN treatment. A representative set of data from two biological repeats is shown. Log-rank test was used for statistics: ***p < 0.0001 compared with WT (EtOH), ^###^*p* < 0.0001 compared with WRN-KD (EtOH). **h**, **i** Effects of NMN (**h**) and NR (**i**) on gut damage-induced proliferating intestinal stem cells (pH3^+^, ISCs) in adult D7 flies. (*n* = 10 flies for each condition, two biological repeats). One-way ANOVA using Sidak’s post-hoc test. Data are shown in mean ± S.E.M. *p *<* 0.05, **p *<* 0.01, ***p *<* 0.001.

Given that NAD^+^ replenishment improves mitochondrial parameters in human WS cells, we further examined whether it increased lifespan in the *wrn-1(gk99) C. elegans*. We treated *wrn-1(gk99)* worms and WT N2 worms with NR (1 mM) or NMN (1 mM) from the L4 stage, and measured lifespan and healthspan. NR increased the lifespan in the N2 worms by 10% (Supplementary Table 4b), in line with previous studies^21,29,37^. The NAD^+^ level was 38% lower in *wrn-1(gk99)* than N2, and NR or NMN treatment increased organismal NAD^+^ 2.2 times compared with vehicle-treated groups (Fig. 3a). Interestingly, we found that NR or NMN dramatically extended the mean lifespan of the *wrn-1(gk99)* worms from 13.9 days to 18.1 days (NR) or 19.8 days (NMN), almost to the lifespan of untreated N2 worms (20.6 days) (Fig. 3b, c and quantification in Supplementary Table 4b). NR or NMN treatment also significantly improved healthspan in the *wrn-1(gk99)* worms, as detected by increased pharyngeal pumping at both adult day (D) 4 and D6 (Fig. 3d). There was no difference in maximum velocity of movement in the worms between genotypes or after NR or NMN treatment (Supplementary Fig. 5a). One hallmark of aging is stem cell dysfunction, which occurs prematurely in human WS cells^5^. Thus, we further examined the number of germ line-localized mitotic cells^38^. The vehicle-treated *wrn-1(gk99)* worms had 18% fewer mitotic cells than N2 worms at D6. After NR treatment, the number of cells increased from 75 cells/worm to 132/worm in *wrn-1(gk99)* worms (Fig. 3e, and a representative set of images in Supplementary Fig. 5b). The number of proliferating cells can be quantified by staining for phosphorylated Histone 3 (pH3)^39^. When worms were treated with 90 Gy of γ-radiation to induce genomic stress, there were less proliferative cells in the *wrn-1(gk99)* worms than in the N2 worms. While NR treatment had no significant effect on the numbers of pH3^+^ cells in N2 worms, it dramatically increased pH3^+^ cells in *wrn-1(gk99)* worms by more than 3-folds (Fig. 3f, and a representative set of images in Supplementary Fig. 5c). Collectively, the data suggest that NAD^+^ repletion extends lifespan and healthspan, and improves the number and proliferative potency of mitotic cells in *wrn-1(gk99) C. elegans*.

Encouraged by the dramatic improvements of lifespan and healthspan by NAD^+^ precursors in the *wrn-1(gk99)* worms, we wondered whether this benefit was conserved across species. We thus examined a *Drosophila melanogaster* model of WS through RU486-dependent induction of RNAi knockdown of *Wrnexo*, hereafter termed *Wrnexo*^*RNAi*^ flies^40^. RU486 treatment induced 60% organismal knockdown of the *Wrn* mRNA (Supplementary Fig. 6c). Compared with WT control (veh) flies, *Wrnexo*^*RNAi*^ flies had a significantly shorter lifespan (Fig. 3g). Importantly, treatment with the NAD^+^ precursor NMN in the *Drosophila* food significantly extended the lifespan in both WT and *Wrnexo*^*RNAi*^ flies, indicating a role of NAD^+^ in maintaining lifespan (Fig. 3g). We also validated and explored NAD^+^-dependent restoration of stem cell function in the *Wrnexo*^*RNAi*^ flies. In response to injury from pathogenic bacteria, *Drosophila* intestinal stem cells (ISCs) proliferate to regenerate the intestine^39^. Using a stem-cell specific inducible driver line (w;5961GS;UASnlsGFP), *Wrnexo* was specifically knocked down in the gut stem cells but not in other gut cells (e.g., enterocytes). The major aim was to avoid possible non-autonomous effects to the ISCs by other gut cells and tissues. In *Wrnexo*^*RNAi*^ flies, infection failed to induce a proliferative response measured by pH3 positive cells (Fig. 3h, i). This effect could be rescued by NR or NMN treatment during the period of RNAi, which had no effect on WT controls (Fig. 3h, i and Supplementary Fig. 6a, b). The *Wrnexo* KD-induced reduction of proliferation in ISCs is not due to loss of stem cells in *Wrnexo*^*RNAi*^ flies, as tested by examining the expression of GFP in the total bunched expressing gut cells (driven by 5961GS) (Supplementary Fig. 6d). Collectively, supplementation with NAD^+^ precursors improved both lifespan and the proliferative potential of ISCs in the WS flies, suggesting a major role of NAD^+^ in healthy longevity.

If NAD^+^ depletion is a major driver of accelerated aging in WS, supplementation with different NAD^+^ precursors^41^ should show similar beneficial effects. Indeed, NMN (1 mM) treatment gave similar anti-aging benefits in *wrn-1(gk99)* as did NR treatment (Fig. 3a–d, and Supplementary Table 4c). NAD^+^ alone also extended the lifespan in the *wrn-1(gk99)* worms (Supplementary Table 4c). NAM treatment extended the lifespan one more day but this did not reach statistical significance (Supplementary Table 4c). We further explored the effect of treatment with NAD^+^ precursors at different ages on lifespan in *wrn-1(gk99)*. Exposure to NR beginning at the egg, L4 stage (last developmental stage), or young adult (D3) stages all had similar effects in extending lifespan. However, the benefit of NR treatment was significantly reduced when exposure began at D5 (Supplementary Fig. 5d–i, and quantifications in Supplementary Table 4d, e). Taken together, these data suggest that NAD^+^ depletion is a driver of accelerated aging in WS, and that NAD^+^ augmentation extends lifespan and healthspan in both worm and fly models of WS.

### Restoration of impaired mitophagy by NAD^+^ repletion in WS

**Fig. 4:**
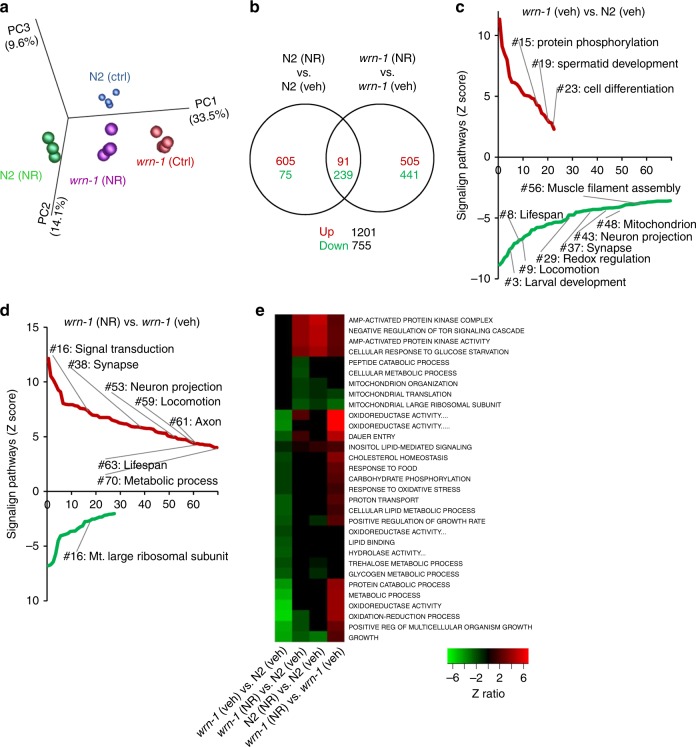
NAD^+^ replenishment normalizes the transcriptomic profiles of WRN. The *wrn-1(gk99)* and the N2 worms were treated with NR (1 mM) from the L4 stage, followed by changing to fresh drug plate on adult Day4. The worms were collected on adult D7 for transcriptomic analysis. **a** Principal component analysis (PCA) revealed separation between N2 (veh) and *wrn-1(gk99)* (veh), while NR treatment led to a normalization of the *wrn-1(gk99)* transcriptomic profile closer to the N2 (veh). **b** Venn diagram of transcriptomic results showing that NR induced changes of genes between [N2 (NR) vs. N2 (veh.)] vs. [*wrn-1* (NR) vs. *wrn-1* (veh.)]. **c**, **d** Gene-set-enrichment analysis demonstrates upregulated and downregulated signaling pathways (GO pathways) in the D7 N2 and the *wrn-1(gk99)* worms treated with/without NR (1 mM from L4). The GO terms were ranked on the basis of enrichment scores. The upregulated GO pathways and downregulated GO pathways summarized separated, with a whole list of changes GO terms shown in Supplementary Table 3. **e** Heat map data showing changes of the GO terms related to mitochondrial function among four different comparisons.

To explore the cellular and molecular mechanisms of WRN and NAD^+^ in aging, we measured whole genome gene expression of N2 and *wrn-1(gk99)* worms with/without NR (from L4, using 1 mM NR) (Fig. 4, Supplementary Fig. 7a, b). Principal component analysis (PCA) revealed a separation between N2 (veh) and *wrn-1(gk99)* (veh), while NR treatment led to a shift of the *wrn-1(gk99)* transcriptomic profile towards the N2 (veh) profile (Fig. 4a). GO term analysis indicated changes in the *wrn-1(gk99)* worms in multiple pathways related to development, metabolism, and ageing, such as larval development, redox regulation and mitochondria, lifespan (Fig. 4b, c), supporting the use of *wrn-1(gk99)* as a model of WS^27^. Interestingly, microarray analysis revealed several changes in GO terms related to fat metabolism (Supplementary Fig. 7b) related to the various metabolism-associated deficiencies reported in human WS patients^42^. NR treatment changed the transcriptomic profile of both N2 and *wrn-1* worms (summarized in Fig. 4b) and increased over 50 GO terms in the N2 worms, including AMPK, mTOR inhibition (which increases autophagy) and the endoplasmic reticulum unfolded protein response (Fig. 4e, Supplementary Fig. 7a). These GO terms are related to energy expenditure, autophagy, cellular stress and aging confirming previous published effects of NAD^+^ precursors supplementation in both *C. elegans* and mice^21,23,29,43,44^. Interestingly, NR treatment changed many GO terms in the *wrn-1(gk99)* worms, especially pathways related to signal transduction, lifespan, and metabolic process (Fig. 4d, e and Supplementary Fig. 7b). Consistent with impaired mitochondrial function by WRN-1 dysfunction (Fig. 1k), heat map analysis of the GO terms suggested a reduction of several mitochondria-related pathways, including oxidoreductase activity pathways, metabolic process, cellular lipid metabolic process, among others; NR treatment activated many of these pathways in the *wrn-1(gk99)* worms (Fig. 4e). Collectively, our transcriptomic analysis suggests that WRN dysfunction impacts metabolism, autophagy, cellular stress responses, development and aging in the *wrn-1(gk99)* worms. NAD^+^ repletion normalizes many of these pathways at the transcript level, supporting the lifespan prolonging effects of NAD^+^ precursors supplementation observed in the animal model of WS. Additionally, the results suggest that cellular stress and maintenance responses as well as metabolic functions are central in WS.

**Fig. 5:**
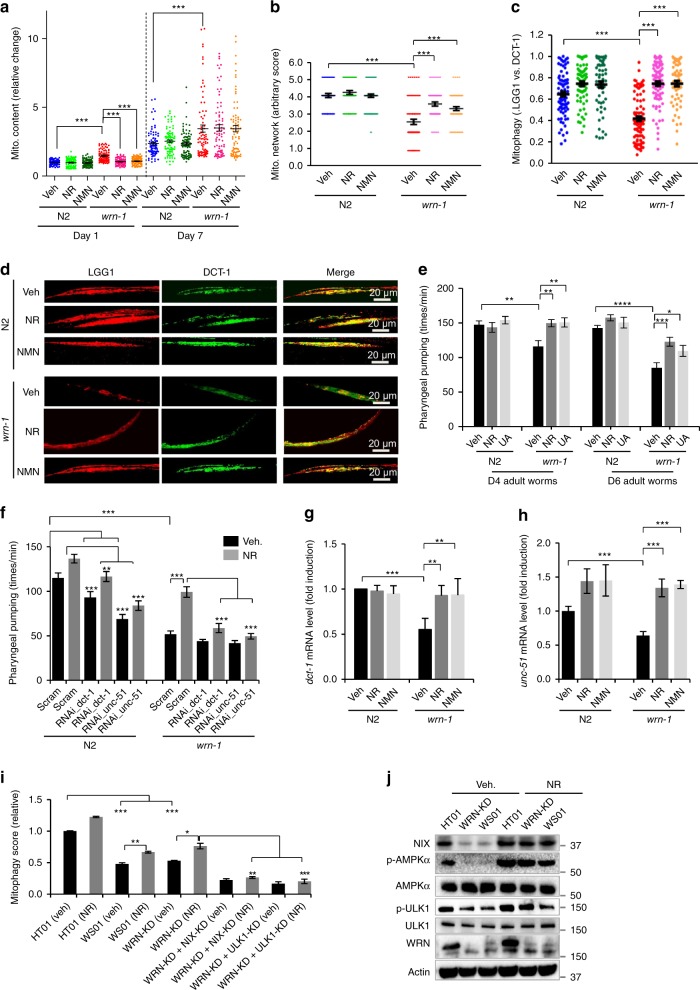
NAD^+^ ameliorates premature aging in WS through DCT-1 and ULK1-dependent mitophagy. **a** Mitochondrial content was evaluated by quantifying MitoTracker green pixels of stained N2 and *wrn-1*(gk99) worms at adult D1 and D7 (*n* = 80 worms; Two-way ANOVA). values pooled from three independent biological repeats. Data from Vehicle-treated worms are also used in Fig. 1h. **b** Quantified scores of muscle mitochondrial morphology of adult D7 N2 and *wrn-1*(*gk99*) worms. A *myo-3::gfp* reporter gene was expressed in body wall muscles. (*n* = 20 worms/group; Two-way ANOVA). **c**, **d** Relative mitophagy rate in adult D7 N2 and *wrn-1*(*gk99*) worms. Mitophagy events were calculated as the co-localization between DsRed::LGG-1 and DCT-1::GFP in muscle cells. (*n* = 20 worms for each condition; Two-way ANOVA). **e** Effects of NR or UA treatment on pharyngeal pumping in N2 and the *wrn-1*(*gk99*) worms at adult D4 and D6. (*n* = 3 biologically independent experiments with 10–20 worms for each condition; Two-way ANOVA). **f** Pharyngeal pumping rates in adult D7 worms of designated groups. (*n* = 20 worms/group; Two-way ANOVA). **g**, **h** mRNA levels of *dct-1* (**d**) and *unc-51* (**e**) in adult D7 worms. (*n* = 3 biologically independent experiments; Two-way ANOVA). **i** Flow cytometry quantification of relative mitophagy incidence in HT01, WS01, and WRN-KD cells under different conditions. For siRNA control, siRNA-vector was added cells. (*n* = 3 biologically independent experiments; Two-way ANOVA). **j** Western blot data showing changes of expression of designated proteins. Source data are provided as a Source Data file. Two-way ANOVA followed by Tukey’s post-hoc tests. Data are shown in mean ± S.E.M. *p *<* 0.05, **p *<* 0.01, ***p *<* 0.001.

Mitochondrial autophagy, termed mitophagy, is the process of clearance of damaged/superfluous mitochondria. It plays a fundamental role in energy expenditure, stem cell rejuvenation, neuroprotection, and healthy aging^14,29^. Our data in WS patients and in the *C. elegans* model of WS detect mitochondrial alterations and accumulation of damaged mitochondria (Fig. 1), and we speculated that these could be caused by impaired mitophagy. To test this hypothesis, we crossed the *wrn-1(gk99)* worms with a mitophagy reporter worm strain to visualize the co-localization of LGG-1 (the worm homolog of mammalian LC3) and DCT-1 (the worm homolog of mammalian NIX/BNIP3L), a well-established indicator of mitophagy initiation^10,21^. Supporting an inducible effect of NAD^+^ supplementation on mitophagy, NR or NMN treatment decreased mitochondrial content in the *wrn-1(gk99)* adult day 1 worms and decreased the mitochondrial membrane potential (MMP) at adult day 7 (Fig. 5a and Supplementary Fig. 8i). Lower MMP normally facilitates accumulation of full-length PINK1 on the outer mitochondrial membrane to activate mitophagy^29,45^. The basal level of mitophagy in muscle cells of the *wrn-1(gk99)* worms was 41% lower than in N2 worms, while two independent NAD^+^ replenishment strategies (NR, NMN) restored mitophagy in *wrn-1(gk99)* to that of N2 (Fig. 5c, d). To further confirm whether mitophagy induction is sufficient to rescue WS in the worm model, we treated *wrn-1(gk99)* worms with a mitophagy specific inducer Urolithin A (UA)^46^. UA improved pharyngeal pumping of the *wrn-1(gk99)* worms similar to the effect of NR (Fig. 5e), and extended the lifespan of the *wrn-1(gk99)* worms (Supplementary Table 4f). We confirmed that UA is a specific mitophagy inducer since siRNA knock down of *dct-1* eliminated UA-dependent extension of lifespan and healthspan (pharyngeal pumping) in the *wrn-1(gk99);dct-1*(RNAi) worms (Fig. 5f, Supplementary Fig. 7c, d, and Supplementary Table 4a). Compared with NR, UA had a smaller effect on lifespan extension in the *wrn-1(gk99)* worms (Supplementary Table 4f), indicating the involvement of mitophagy-independent, additional pathways in NR-contributed lifespan extension. Possible additional NAD^+^-dependent longevity pathways include the NAD^+^-SIRT1-PGC1α-dependent mitochondrial biogenesis, the NAD^+^/sirtuins-dependent metabolism, and the NAD^+^-dependent DNA repair pathways^21,29,47^.

To investigate which mitophagy-related proteins were involved in NAD^+^-induced mitophagy and related healthspan improvement (e.g., pharyngeal pumping), we knocked down known mitophagy genes in the worms through RNAi feeding and analyzed the changes in pharyngeal pumping. Two mitophagy-associated genes, *dct-1* (mammalian *NIX*) and *unc-51* (mammalian *ULK1*), were involved in NAD^+^-induced improvement in pharyngeal pumping in both N2 and the *wrn-1(gk99)* worms (Fig. 5f). Indeed, the mRNA levels of *dct-1* and *unc-51* were lower in the *wrn-1(gk99)* worms, and two NAD^+^ replenishment strategies significantly increased *dct-1* and *unc-51* expression (Fig. 5g, h). NAD^+^ replenishment had no detectable transcriptional effect on other mitophagy-associated genes, such as *pink1* and *pdr-1* (*Parkin* in mammals), although WRN dysfunction also decreased *pink-1* mRNA (Supplementary Fig. 8a). The two NAD^+^ replenishment strategies improved the muscle mitochondrial network (Fig. 5b and Supplementary Fig. 8b) and mitochondrial size (Supplementary Fig. 8c, d). The benefit of NAD^+^ replenishment in mitochondrial morphology and parameters in the *wrn-1(gk99)* worms may also be through its regulation of mitochondrial fusion and fission related genes (such as *fzo-1*, *opa-1*, and *drp-1*) (Supplementary Fig. 8e) and the induction of mitochondrial antioxidant superoxide dismutase 3 (SOD3)^48^ (Supplementary Fig. 8f). These results suggest that NAD^+^ can coordinate turnover and dynamics of mitochondria. We further confirmed that mammalian NIX and ULK1 were necessary for NAD^+^-dependent mitophagy in WS1 and WRN-KD cells. In both WS01 and WRN-KD cells, NR treatment increased the level of mitophagy in a NIX or ULK-1 dependent manner (Fig. 5i). It has been shown that AMPK regulates energy expenditure through phosphorylation of ULK1 at Ser555^43^. Indeed, AMPK activity, as detected using antibodies specific for phosphorylated forms of AMPKα (p-Thr127) and its downstream target p-ULK1 (p-Ser555), was lower in WRN deficient cells, and NAD^+^ replenishment restored AMPKα activity (increased level of phosphorylated AMPKα, p-AMPKα) (Fig. 5j, and quantification in Supplementary Fig. 8h). We extended our analysis from protein expressions to the imaging of mitophagic events in the WS01 primary cells by siRNA knock down of *ULK1*, *AMPK*, or both. While AMPK, ULK1, or AMPK + ULK1 contributed to NR-induced mitophagy, other NAD^+^-dependent mitophagy pathways were likely involved^24^ since knock down of *AMPK*, *ULK1*, or *AMPK* + *ULK1* was unable to fully ablate NR-induced mitophagy (Supplementary Fig. 9). In the WRN-KD cells there were no impairments of the macro-autophagic machinery, and the basal level of macro-autophagy was increased (increased levels of p62 and LC3 II/I) (Supplementary Fig. 8g), in line with previous work^49^. It should be noticed that upregulation of autophagic proteins does not always correlate with increased autophagy events^50,51^. However, it appears to be a common phenomenon in DNA repair-deficient premature ageing diseases: while there is an increase of autophagy through mTOR inhibition and ROS-regulated autophagy induction, there is impaired mitophagy (possibly due to higher membrane potential and larger mitochondria) in many DNA repair deficient premature ageing diseases^52–54^. In summary, our data from *C. elegans* and human cells consistently indicate impaired mitophagy in WS, and that NAD^+^ replenishment restores mitophagy by a mechanism involving DCT-1 (NIX) and UNC-51 (ULK1).

### NAD^+^ restores fat metabolism in the WS worms

**Fig. 6:**
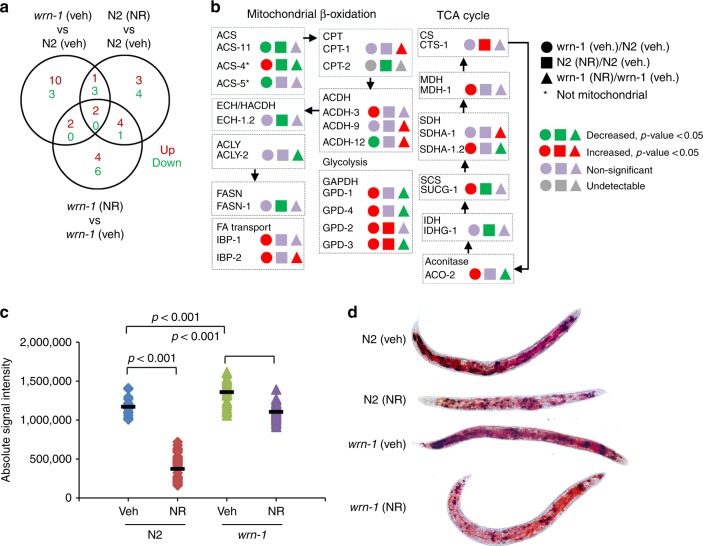
Effects of NAD^+^ replenishment on fat metabolism in adult Day 7 *wrn-1 C. elegans*. We performed systematic proteomic analysis using mass spectrometry with samples from the whole-body tissues from adult D7 worms (*n* = 4 biologically independent experiments). We then comprehensively evaluated the changes of fat-metabolism-related molecular pathways, including mitochondrial β-oxidation, peroxisomal β-oxidation, lipolysis, glycolysis, fatty acid transport, as well as fatty acid desaturation and elongation^57^. **a**, **b** A summary of significantly altered proteins involved in fat metabolism are shown. Abbreviations used: ACDH: acyl-CoA dehydrogenase, ACLY: ATP citrate lyase, ACS: acyl-CoA synthetase, CPT: carnitine palmitoyl transferase, CS: citrate synthase, ECH: enoyl-CoA hydratase, FASN: fatty-acid synthase, IDH: isocitrate dehydrogenase, PFK: phospho-fructo-kinase, SCS: succinyl-CoA synthetase, SDH: succinate dehydrogenase, MDH: malate dehydrogenase. **c**, **d** Changes of fat content in the adult D7 worms treated with/without NR from L4. Oil Red staining was used for imaging with quantification using ImageJ. *n* = 30 worms/group (Two-way ANOVA). For **c**, the bars stand the independent value of mean within each group.

Mitophagy-dependent mitochondrial quality control regulates fat metabolism^55,56^. We next determined whether NAD^+^ replenishment could improve fat metabolism in the *wrn-1(gk99)* worms. We performed transcriptomic and metabolomic analysis using whole bodies from adult D1 and adult D7 worms. At the transcriptional level, WRN-1 dysfunction inhibited almost all of the lipid metabolism-related pathways, including cellular lipid metabolic process, lipid transport, and lipid transporter activity (Supplementary Fig. 7b). NR treatment increased the activity of some of these pathways (Supplementary Fig. 7b). We used our metabolomic data to evaluate the changes in fat-metabolism-related molecular pathways^57,58^. Within the same age, many fat-metabolism/catabolism-related proteins were differentially expressed in the *wrn-1(gk99)* worms compared to the N2 worms. Figure 6a shows a Venn diagram of the significantly changed proteins, Fig. 6b shows the significantly changed proteins, and Supplementary Table 3 shows the entire set of proteins analyzed. Interestingly, the ACDH proteins are linked to energy metabolism and CR-related longevity in *C. elegans*^59^. We finally asked whether NR could affect lipid storage at the organismal level in the *wrn-1*(*gk99*) worms. We stained lipids using oil red in Day 7 worms. While WRN-1 dysfunction significantly increased the amount of whole-body lipid content (*wrn-1* veh. vs. N2 veh), NR treatment decreased whole organismal lipid levels to 80% in the *wrn-1*(*gk99*) worms (*wrn-1* NR vs. *wrn-1* veh) (Fig. 6c, d). However, we did not detect any increase of OCR in the NR-supplemented young (adult day 2) and old (adult day 10) *wrn-1* worms (Supplementary Fig. 1). Collectively, our combined transcriptomic, metabolomic, and fat staining data support a role of WRN in fat metabolism, and NR treatment may partially improve the abnormal fat metabolism in the *wrn-1(gk99)* worms.

### WRN dysfunction affects additional aging-related pathways

**Fig. 7:**
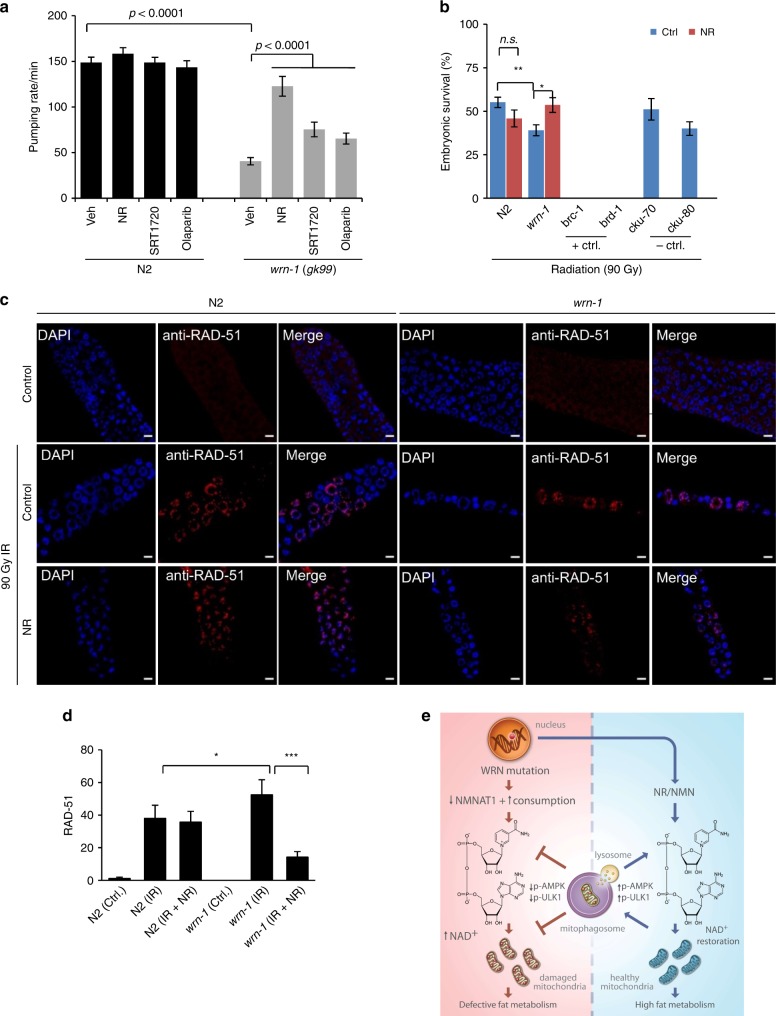
Effects of PARP1, sirtuins on the healthspan, and of NR on DNA repair in the *wrn-1* worms. **a** Pumping rate of the adult D6 worms. Data are shown in mean ± S.E.M (*n* = 20 worms per condition). For all the experiments, worms from L4 stage were exposed to vehicle control, the NAD^+^ precursors NR and NMN (both at 1 mM), a SIRT1activator SRT1720 (10 µM), or a PARP inhibitor Olaparib (500 nM). Two-way ANOVA followed by Tukey’s post-hoc tests was used for data analysis. **b** Embryonic homologous recombination (HR) capacity was measured by scoring the percent survival of early stage embryos after irradiation with 90 Gy. Two HR mutants, *brc-1(tm1145)* and *brd-1(dw1)*, were used as positive controls, while two NHEJ mutants, *cku-70(tm1524)* and *cku-80(ok861)*, were negative controls^21^. The results were from four biological replicates, including 400–900 worms for each condition. Data shown are mean ± S.E.M. (One-way ANOVA). **c**, **d** Changes of RAD-51 signals in the mitotic region of designated groups of worms. Worms were exposed to 90 Gy ionizing radiation and germlines were isolated 24 h post-irradiation. Immunostaining of DAPI and RAD51 in the germlines were performed using standard protocols (Two-way ANOVA was used for data analysis with *p *<* 0.05, **p *<* 0.01, ***p *<* 0.001). For (**c**), scale bars, 10 µm. **e** Working model. We propose *WRN* mutation leads to cellular NAD^+^ reduction through the down-regulation of the NAD^+^ synthetic enzyme NMNAT1 and upregulation of cellular NAD^+^ consumption (e.g., by PARPs). Cellular NAD^+^ depletion impairs mitophagy through reduction of the activities of p-AMPK and p-ULK1, two upstream proteins which regulate autophagy/mitophagy. This results in accumulation of damaged mitochondria and impaired mitochondrial homeostasis. In combination with the defects in this NAD^+^-AMPK-ULK1-mitophagy pathway and other WRN-dependent cellular processes, such as DNA repair, they drive defective metabolism and accelerated aging. NAD^+^ augmentation through NAD^+^ precursors, such as NR (nicotinamide riboside) and NMN (nicotinamide mononucleotide) alleviate *WRN mutation*-induced pathological features in both *C. elegans* and *Drosophila* models of WS and in primary human cells from WS patients.

As NAD^+^ depletion may drive accelerated aging in WS, we aimed to determine underlying molecular mechanisms. NAD^+^ depletion can affect the activity of NAD^+^ consuming enzymes, such as PARPs, sirtuins and CD38^15,16,37^ (Supplementary Fig. 3o). We were especially interested in PARPs, which are often activated in response to DNA damage and can lead to NAD^+^ depletion. This would limit the activities of other NAD^+^-dependent enzymes, such as sirtuins, which play a major role in metabolism and health^15,16,37^. We asked whether PARP inhibition was able to decrease disease phenotypes. Olaparib, a specific PARP inhibitor, improved both lifespan (Supplementary Table 4b) and healthspan (pumping rate, Fig. 7a) in the *wrn-1(gk99)* worms, indicating that PARP-dependent NAD^+^ consumption contributes to the phenotype of this *C. elegans* WS model. A SIRT1 activator, SRT1720, showed effects similar to Olaparib. Compared with *wrn-1(gk99)* (veh, mean lifespan 13.9 days), both Olaparib (15.0 days) and SIRT1720 (16.5 days) slightly extended lifespan, but less than NR (18.1 days) and NMN (19.8 days) (Supplementary Table 4b). Collectively, these results suggest that NAD^+^ depletion may affect PARylation and sirtuin pathways in addition to mitophagy pathways.

Because DNA repair deficiency is prominent in WS^2,60^, we explored whether NR was able to improve DNA repair in WS. WRN mutation causes a defect in homologous recombination (HR)^61^, we thus performed an embryonic HR capacity assay by exposing early stage embryos to ionizing radiation (90 Gy)^62^. Compared with N2 embryos, the *wrn-1(gk99)* embryos were more sensitive to ionizing radiation (90 Gy) as indicated by less embryonic survival (Fig. 7b). While NR had no significant effect of HR in the N2 embryos, it significantly improved embryonic survival in the *wrn-1(gk99)* embryos (Fig. 7b). WRN plays a role in RAD51-dependent HR^61^, and we thus checked RAD-51 filament formation in the *wrn-1(gk99)* germline after 90 Gy ionizing radiation. Compared with N2 worms, there were increased numbers of RAD-51-positive foci/mitotic region in the *wrn-1(gk99)* worms indicating increased DNA damage (Fig. 7c, d). Consistently, NR had no significant effect in the N2 worms, but it dramatically decreased the numbers of RAD-51 cells in the mitotic region in the *wrn-1(gk99)* worms (Fig. 7c, d). Altogether, these data indicate that NR increases HR DNA repair in the WS *C. elegans*.

## Discussion

We report that WS is associated with a significant mitochondrial dysfunction, mainly manifested as defective mitophagy. This is reflected in lower NAD^+^ levels across species from worms to humans. NAD^+^ supplementation improves mitochondrial function and other age-related metabolic outcomes. Mitochondrial disease can manifest itself in multiple clinical outcomes amongst which neurodegeneration and impaired metabolism are common^9,63^. Strikingly, our microarray data strongly suggest important roles of WRN in neuronal development and neuroplasticity in *C. elegans*. WS patients do not appear to have significantly increased neurodegeneration. However, recent studies suggest that brain atrophy occurs in WS patients, and a linkage between *WRN* polymorphisms and several brain disorders has been reported^60,64^. In mouse brain, there was an age-dependent increase of *Wrn* as well as of WRN-related pathway transcriptomes^65^. Further studies on how WRN coordinates mitochondrial function and neuronal health are warranted. We also find defects in fat metabolism, which may contribute to diabetes and aggravate features of WS. These findings may also relate to mitochondrial dysfunction and defective mitophagy, and we find that NAD^+^ supplementation improves them. Thus, in considering mechanisms and intervention, mitochondrial health may be a new target in WS (Fig. 7e).

Exploration of the interconnected networks between NAD^+^ depletion and WRN dysfunction provides insight into the aging process. In addition to its classical roles in energy metabolism, NAD^+^ is emerging as a fundamental element in health and aging as evidenced in a series of animal and human studies as well as its strong associations with the hallmarks of aging^15,16^. This present study further links NAD^+^ depletion as a major driver of accelerated aging in two animal models of WS (*C. elegans* and *Drosophila*), consolidating its importance in longevity. Our systematic transcriptomic and metabolomic analyses in combination with a series of functional analysis in the *wrn-1(gk99) C. elegans* suggest that NAD^+^ repletion improves fat metabolism and healthspan as well as lifespan. Our data suggest an important role for stimulation of mitophagy among the beneficial effects of NAD^+^ replenishment on WS phenotypes in patient cells and worm and fly models. Additionally, the differential contributions of WRN-exonuclease and WRN-helicase in organismal aging are not clear^2,66^. The exonuclease (WRN-exo) activity of WRN makes it unique from other RecQ helicase family members. The *C. elegans* WRN only has the helicase domain (no WRN-exo) while the *Drosophila* WRN only contains the WRN-exo domain (no helicase domain)^27,40^. Our data indicate that both WRN-exo and WRN-helicase are necessary for organismal lifespan and healthspan, including stem cell function^30^. In addition to its major roles in double-strand break DNA repair and in base excision repair^2^, WRN has also shown to be a transcription factor^35,67^. In that regard, we find a role for WRN in NAD^+^ biosynthesis via its regulation of NMNAT1 at the transcriptional level. In addition to the salvage pathway, on which we have focused in this study, the de novo biosynthesis (kynurenine pathway) and the Preiss-Handler pathway are additional pathways for cellular NAD^+^ synthesis^15,17^. Studies show that NR mainly works through the salvage pathway^32,68^.

Here, we provide molecular insight into how NAD^+^ regulates mitophagy. Elevation of intracellular NAD^+^ levels enhances the activity of the NAD^+^-dependent sirtuins, which deacetylate proteins involved in DNA repair and mitochondrial stress resistance^16,21^. They also regulate autophagy/mitophagy, e.g., the NAD^+^-dependent SIRT1 regulates autophagy/mitophagy through an acetylation/deacetylation-dependent nucleo-cytoplasmic transport and activation of Atg8 (LC3)^69^ as well as the physical binding and deacetylation of several essential components of the autophagy machinery, including Atg5, Atg7 and Atg8^70^. Further, the mitochondrial Sirtuins (SIRT3–5) also regulate mitophagy^71–73^. Recently, we reported that NAD^+^-dependent SIRT1 regulates mitophagy through a DAF-16-DCT1 pathway^21^. As a key metabolic sensor/regulator, AMPK is linked to NAD^+^ metabolism, SIRT1 activity, and the phosphorylation of the autophagy / mitophagy protein ULK1 at Ser555^43,63^. Here we provide further evidence that a central node in the pathway is WRN’s regulation of mitophagy through the NAD^+^/Sirtuins-AMPK-ULK1 pathway. It has been shown^10,14,74^. that autophagy/mitophagy is necessary to maintain quiescence and stemness of stem cells by clearing both active and damaged mitochondria^74^. Interestingly, we show impaired stem cell proliferation in animal models of WS, while NAD^+^ supplementation was able to improve the proliferation potency of stem cells. This study also suggest an ‘increased macro-autophagy and an impaired mitophagy’ in WS, a phenomenon, which is seen in other DNA repair accelerated aging models including Cockayne syndrome, Ataxia telangiectasia, Xeroderma pigmentosum, and possibly Fanconi anemia^21,47,53,54^.

Overall, our study uncovers a previously unknown regulatory mechanism that connect the DNA repair protein WRN to the anti-aging molecule NAD^+^. They function together to preserve energy homeostasis through orchestrating the turnover (mitophagy) and functionality of mitochondria. In *C. elegans*, increased NAD^+^ regulates mitochondrial homeostasis through DCT-1 and ULK-1-dependent mitophagy, leading to a healthier mitochondrial pool which can more efficiently produce ATP while reducing ROS production. Further studies on the interconnected networks between WRN, NAD^+^, and mitophagy, and the roles of these networks in energy expenditure and healthy aging, are of fundamental importance and may suggest clinical applications.

## Methods

### Cell Culture, RNAi knockdown, and mitochondrial parameters

The primary fibroblast cell lines HT01 (#AG09599) and the WS01 (#AG03141) cells were acquired from Coriell Institute. The WRN-KD cells were siRNA knockdown in HT01 cells using WRN human siRNA ologo duplex (CAT#: SR322215, Origene). Briefly, siRNAs were incubated in Optimem with 4 ml RNA Interferin (siRNA transfection reagent, Polyplus) per 1 ng RNA for 15 min and added to complete media for a final concentration of 30 nM siRNA. After 3-day incubation, cells were applied for further experiments. Knockdown efficiency was examined by western blot. All other primary human fibroblasts (detailed in Supplementary Table 1) and blood samples (detailed in Supplementary Table 1) were from Chiba University. The protocols (SEI973 and SEI974) were approved by the Bioethics committee of the Chiba University Graduate School of Medicine, and was conducted in full compliance with the Declaration of Helsinki. Participants provided written informed consents. The mouse embryonic fibroblasts (MEFs) were generated from *Wrn−/−* embryos with MEFS from the wild type littermates as controls. All cells were maintained in GIBCO MEM medium supplemented with 15% FBS, 1% P&S, 1x Vitamin C, 1x Glutamine, and 1x NEAA, and grown in 20% O_2_/5% CO_2_ at 37 °C. Flow cytometry was used mitochondrial parameters, electron microscopy for mitochondrial morphology, an XFe96 for mitochondrial oxygen consumption rate (OCR), and a luminescent assay for ATP quantification. First, human primary cells (HT01, WRN-KD, WS01) or MEFs (WT and Wrn^−*/−*^ cells) were stained with designated dyes followed by flow cytometry to quantify relative mitochondrial ROS (using mitoSOX dye 3 μM for 30 min), mitochondrial membrane potential/MMP (TMRM dye, 10 nM for 15 min), or relative mitochondrial content (MitoTracker Green, 50 nm for 30 min)^54^. All reagents were from Life Technologies. Data was analyzed using FCS Express 4 software (De Novo Software). ATP levels in the primary human fibroblasts were examined using a commercial kit (abcam #ab113849). Cells of 6–10 passages were used for the experiment.

### Mitophagy detection in human primary cells

A commercial kit (Dojindo Laboratories) was used to detect mitophagy in cultured primary fibroblasts and *WRN*-KD cells^23,75^. In brief, Mtphagy dye (Dojindo Laboratories) was added to the cells, where after it accumulates in the intact mitochondria. A decrease in pH (when mitochondria are localized to lysosomes) results in a high fluorescence signal from the dye, which was used to sort the cells using flow cytometry as previously described^23,75^. For microscopic observation, in brief, human cells were treated with Mtphagy dye for 30 min, then after the supernatant was discarded, cells were treated with NR (1 mM) for 24 h, and then the lysosomal dye was incubated for 30 min. Mitophagy events were imaged under the microscope. For the ULK1 and AMPK knockdown in human cells, we use ULK1 siRNA (Origene, #SR322391) and AMPK siRNA (Origene, #SR321409) transient transfection in WS01 fibroblasts.

### Mice and mitochondrial functions

Mice carrying *Wrn*^−*/−*^ allele^30^ were maintained under standard laboratory conditions at the NIA with free access to water and standard diets. All procedures were approved by the Animal Care and Use Committee of the Intramural Research Program of the National Institute on Aging (NIA), in accordance with the National Research Council’s Guide for the Care and Use of Laboratory Animals. Our protocol 361-LMG received an ethical approval as a part of the review process. High resolution respirometry was performed on the Oxygraph-2k (O2k, OROBOROS Instruments, Innsbruck, Austria) according to previous report^76^. Mice were sacrificed by cervical dislocation and brain, heart and liver tissues were rapidly dissected and homogenized by 10–15 strokes of a 15 mL dounce homogenizer in Mir05 respiration buffer (110 mM sucrose, 60 mM K-lactobionate, 0.5 mM EGTA, 3 mM MgCl2, 20 mM taurine, 10 mM KH2PO4, 20 mM HEPES, and 0.1% BSA essentially fatty acid free, pH 7.1 at 37 °C). Protein was quantified by BCA assay and 1 mg/mL homogenate loaded for respiration measurements at 37 °C. Malate (2.5 mM), pyruvate (5 mM) and ADP (2.5 mM) addition results in complex I mediated state 3 respiration. Subsequently, complex I inhibitor rotenone (0.5 μM) was added and then complex II substrate succinate (10 mM) injection initiates complex II mediated state 3 respiration. Complex III inhibitor antimycin A (2 μM) was then added followed by the artificial complex IV substrate ascorbate + TMPD (4 mM + 1 mM, respectively). Fatty acid metabolism of the heart homogenate was also performed using malate plus palmitoyl-carnitine (5 mM each) followed by state 3 respiration initiate with 2.5 mM ADP.

### Fly lines, lifespan and stem cell proliferation assays

To temporally control WRN knockdown, we used GeneSwitch lines expressing an inactivated form of Gal4 that is activated in the presence of the drug RU486^77^. We used w;DaGeneSwitch-Gal4 for ubiquitous knockdown and w;5961GeneSwitch-Gal4, UAS-nlsGFP/CyO (a gift from Benjamin Ohlstein^78^) for intestinal stem cell-specific knockdown. We used y,v,sc;WrnexoRNAi; (Bloomington line #38297) for knockdown, and w;LacZ RNAi (a gift from Masayuki Miura) as controls for non-specific RNAi effects. Knockdown of Wrnexo was confirmed by qPCR analysis. RNA was purified from single flies (3 flies/genotype) treated with EtOH, RU486 or RU486 and 5 mM NMN for 7 days with Nucleospin RNA purification kit following manufacturer’s protocol (Macherey-Nagel). cDNA was synthesized using iScript cDNA Synthesis kit (BioRad) and qPCR analysis was done with *power* SYBR Green PCR master mix (Thermo Fisher). To confirm knockdown two different WRNexo primer sets were used: WRNexo F1: 5′-GAAAAGAACGGAGATGCTGCC-3′, WRNexo R1: 5′-AGTCACCTCGTTGATCTTGGTC-3′. WRNexo F2: 5′-AGAACGGAGATGCTGCCTTTA-3′, WRNexo R2: 5′-AGTCACCTCGTTGATCTTGGT-3′, Actin was used as household gene with the following primers: Actin F: 5′-TTCCCCTCCATCGTCGGTC-3′ and Actin R: 5′-GATACCACGCTTGGACTGGG-3′. Reaction efficacy was optimized to 90–100% and the relative difference was calculated by the ddCt method^79^.

Flies were maintained at 25 °C on a 12-h light/dark cycle, using the following food recipe: 1 L distilled water, 13 g agar, 22 g molasses, 65 g malt extract, 18 g brewer’s yeast, 80 g corn flour, 10 g soy flour, 6.2 ml propionic acid, and 2 g methyl-p-benzoate in 7.3 ml of EtOH. For treatment food, RU486 was dissolved in the EtOH for 200 μM final concentration. Lifespan experiments were performed as previously described^80^. Briefly, female offspring of DaGeneSwitch and WrnexoRNAi lines were allowed to mate for 2–3 days, then transferred to vials with food ± RU486 and/or NMN at 5 mM final concentration. Up to 70 flies per vial were flipped thrice weekly, with dead flies counted visually. The Kaplan–Meier lifespan curves were generated using Prism. For statistics, multiple condition experiments were evaluated by one-way ANOVA with statistics using Peto’s log-rank test (Cox-Mantel test).

The 5961GeneSwitch-Gal4, UAS-nlsGFP line was crossed to Wrnexo or LacZ RNAi to evaluate stem cell proliferation in response to pathogenic insult. After allowed to mate for 2 days after eclosion female offspring were transferred to food with/without RU486 (200 μM), ±NR supplementation (0.78 mM) or NMN supplementation (5 mM). At six days of age (after four days of RNAi), they were transferred to 5% sucrose ± Erwinia carotovora carotovora 15 from 15 mL of overnight culture. After 24 h, guts were dissected in 1x phosphate-buffered saline (PBS), fixed for 45 min at room temperature (100 mM glutamic acid, 25 mM KCl, 20 mM MgSO_4_, 4 mM sodium phosphate, 1 mM MgCl_2_, and 4% formaldehyde), washed for 1 h at 4 °C (1x PBS, 0.5% bovine serum albumin and 0.1% Triton X-100), and then incubated with rabbit anti-phospho-Histone H3 Ser 10 (Upstate, 1:1000) overnight at 4 °C. Secondary antibody staining was done at room temperature for 2 h, using fluorescent antibody from Jackson Immunoresearch at 1:500. DAPI was used to stain DNA (1:1000). Guts were washed 3 × 10 min after each antibody. pH3 positive cells were counted manually on a Zeiss dissecting fluorescent microscope, with representative images captured on a Zeiss LSM 700 confocal microscope and processed using Adobe Photoshop/Illustrator. The UAS-nlsGFP construct was used to evaluate the total number of intestinal stem cells, to rule out cell death as a factor in the proliferative response. Comparisons between proliferation counts were done by one-way ANOVA, using Sidak’s post-hoc test.

### *C. elegans* strains, and lifespan/healthspan studies

Standard *C. elegans* strain maintenance procedures were followed in all experiments^81^. Nematode rearing temperature was kept at 25 °C, unless noted otherwise. N2: wild type Bristol isolate was from Caenorhabditis Genetics Center and the *wrn-1(gk99)* was a gift from Dr. Hyeon-Sook Koo (Yonsei University, Korea)^27^. RNAi knockdown of designated genes was performed using standard protocol and verified by PCR^21^. Lifespan examination was performed at 25 °C on NGM plates containing 100 μM 5-FudR and seeded with 100 μL E. coli OP50 strain. Worms were scored every day (Urolithin A experiments) or every other day (NR or NMN treatments) and scored as dead when they stopped pharyngeal pumping and were unresponsive to touch. Lifespan experiments were performed with 100–150 worms/group. Kaplan–Meier survival curves of pooled populations were generated and the log-rank test was used for statistics. Swimming and pharyngeal pumping were used for healthspan evaluation following established methods^21^, with 10–30 worms/group (three biological repeats). Pharyngeal pumping was evaluated at adult day 4 and adult 6. Drug or vehicle treatment began at L4 stage, unless noted otherwise. Drugs used were the NAD^+^ precursors NR and NMN (both at 1 mM), the mitophagy inducer Urolithin A (100 μM), a SIRT1 activator SRT1720 (10 μM), and a PARP inhibitor Olaparib (500 nM). Two to seven biological repeats were performed for all experiments. OCR of *C. elegans* was measured using Seahorse XFe96 instrument^75,82^. *wrn-1* and N2 *C. elegans* strains were synchronized by standard egg lay and L4 stage nematodes were transferred to plates +/− NR (1 mM). At days 2 and 10, worms were washed three times with M9 buffer and allowed to digest gut bacteria for 30 min prior to the start of respiration. Worms were plated in XFe96 seahorse plates in M9 buffer (15–30 worms per well). Instrument was started with six measurements of each respiratory state. First, baseline respiration was measured followed by injection of FCCP (10 μM, final concentration) to elicit maximal respiration. Then, sodium azide (40 mM, final concentration) was injected to account for non-mitochondrial respiration. Number of nematodes per well were counted for normalization.

### Quantification of mitotic cells in *C. elegans*

For quantification of mitotic cells, immunostaining was performed^83^. Briefly, worms were treated with NR (1 mM) at L4 stage and germlines were isolated on adult day 1 and adult day 6 worms. Germlines were isolated on poly-l-lysine-coated slides in egg buffer (containing 25 mM HEPES, pH 7.4, 118 mM NaCl, 48 mM KCl, 2 mM CaCl_2_, 2 mM MgCl_2_) supplemented with 0.1% Tween-20 and 0.2 mM levamisol. The slides were mounted with 7 μl mounting solution containing ProLong Gold (Thermo Fisher) and 0.5 μg/ml DAPI (Sigma), followed by imaging in a confocal microscope. Number of mitotic cells was measured using ZEN 2.3 software. The distal edge of the transition zone border was defined as the first cell diameter in which two or more nuclei displayed the characteristic crescent shape.

### *C. elegans* imaging

We performed imaging for mitochondrial network, mitochondrial content, mitophagy levels, and fat staining. To evaluate mitochondrial network in worm muscle cells, a *myo-3::gfp* reporter strain was imaged with Zeiss confocal microscopy, with 5 images/worm and 15 worms/group/experiment. Mitochondrial network was scored in a double-blinded manner on an arbitrary scale from 1 to 5. A score of 5 denotes a perfectly organized mitochondrial network with healthy mitochondria running parallel with the myofilament lattice. For highly fragmented and disorganized mitochondrial network morphology, we gave a score of 19. For mitochondrial content analysis, *wrn-1* and N2 *C. elegans* strains were synchronized by standard egg lay and L4 stage nematodes were transferred to plates containing NR (1 mM), NMN (1 mM), or vehicle control. For MitoTracker staining, worms were cultivated in presence of 125 nM MitoTracker Green FM (#M7514; Life Technologies) at 20 °C for 24 h. Stained and washed worms were immobilized with levamisole before mounting on 2% agarose pads for microscopic examination with a Zeiss AxioImager Z2 epifluorescence microscope. TMRE staining (tetramethylrhodamine, ethyl ester, perchlorate; a dye that accumulates in intact, respiring mitochondria) was conducted as previously described to measure mitochondrial membrane potential^10^. Briefly, animals were grown at 20 °C in the presence of 150 nM TMRE for 24 h. Stained and washed worms were immobilized with levamisole before mounting on 2% agarose pads for microscopic examination with a Zeiss AxioImager Z2 epifluorescence microscope. All images were acquired under the same exposure. Average pixel intensity values were calculated by sampling images of different animals. The mean pixel intensity for each animal were calculated in these images using the ImageJ software (http://rsb.info.nih.gov/ij/). Mean values were compared using Two-way (ANOVA) variance analysis followed by the post-hoc Bonferroni’s multiple comparison test. For each experiment, at least 20–30 animals were examined for each experimental condition. Each assay was repeated at least three times. We used the Prism software package (GraphPad Software) for statistical analyses. Similarly, a MitoTracker Red dye was used for organismal mitochondrial ROS quantification^10^. The mitophagy reporter strain N2;Ex*(pmyo-3::dsred::lgg-1;pdct-1::dct-1::gfp)* (a gift from Dr.Tavernarakis^10^) was crossed with the *wrn-1(gk99)*, followed by evaluation of mitophagy as detailed previously^21^. For imaging of mitophagy signals, we randomly took over 5 images/worm with 15 worms/group/ experiment with at least two biological repeats.

### Fat staining in *C. elegans*

Oil Red O staining was carried out as previously described with some modifications^84,85^. Day 7 old worms (100–200 adults) were^77^ washed three times with PBS then fixed in 200 μl of 1x PBS, 10% PFA and 2XMRWB (KCl 160 mM, NaCl 40 mM, NaEGTA 14 mM, 30 mM PIPES pH 7.4, 0.4 Spermine 1 mM, 1 mM Spermidine, 0.2% beta-mercaptoethanol) buffer for 1 h in an end over end mixing at room temperature. Fixed worms were washed three times with 100 mM Tris-HCl (pH 7.4). After the washes, the worms were incubated with 250 μl 40 mM DTT for 30 min at RT, followed by three washes in 1x PBS and a 15 min incubation with 70% isopropanol. Isopropanol was removed, and 1 ml of 60% Oil-Red-O dye (Sigma-Aldrich Cat. No. O9755) was added. Oil-Red-O solution was prepared by dissolving the dye in isopropanol at 5 mg/ml and equilibrating for several days. The solution was then freshly diluted with 40% water to obtain a 60% stock, allowed to sit 10 min at room temperature and filtered before using to remove insoluble material. Stained animals were incubated overnight with rocking at room temperature. The dye was then removed and 200 μl of 1x PBS with 0.01% TritonX-100 was added. Oil-Red-O absorbs light at 510 nm^85^. Using ImageJ we measured the average pixel intensity for a 40 pixel radius in an area behind the pharynx of each animal. A minimum of 40 animals were measured for each strain.

### Mass spectrometric analysis of PAR levels

For quantitation of PAR levels in *C. elegans*, ~3000 worms were collected at an adult age of 7 days and snap frozen in liquid nitrogen. Thereafter, worm pellets were resuspended in 1 ml 10% TCA and subjected to five freeze-thaw cycles using liquid nitrogen and a 37 °C water bath. Then, samples were centrifuged at 3000 × *g* for 10 min at 4 °C. The TCA pellets were washed twice in 500 μl ice-cold 70% EtOH, air-dried, and stored at −20 °C until further processing. Afterwards PAR was purified and analyzed by mass spectrometry^86,87^.

### Microarray using *C. elegans* samples

N2 and *wrn-1(gk99)* worms were treated with vehicle (H_2_O) or NR (1 mM final concentration) from L4 stage, followed by collection of the worm tissues on adult day 1 and adult day 7 (new drug plates were replaced with on adult day 4). The worms were then washed with M9 buffer three times and flash frozen. The samples were subjected to microarray as detailed previously^21^. Three independent biological samples of the worms were collected for microarray.

### Electron microscopy

EM was used to examine ultrastructure of mitochondrial morphology which was performed by a US Certified Electron Microscopist Dr. J. Bernbaum. 25–30 images were randomly taken for each sample. Quantification of mitochondrial parameters in different cell types was performed using ImageJ plugin ObjectJ (length, diameter, and area). Percentage of damaged mitochondria as well as mitophagic-like events were calculated. All quantifications were performed in a double-bind manner with Two-way ANOVA used for the comparison between multiple groups.

### Metabolomics

Three independent methods were applied for 3 types of purposes as summarized and detailed below. Except human blood samples (one biological sample per person), samples of three biological repeats from human cell culture or the cell culture media from the human cells were used for data collection. The descriptions of all metabolomics methods and data complies were in line with the community requirements.

- For single NAD^+^ detection in the human primary fibroblasts (Fig. 1, three biological repeats of samples) or blood samples (one biological sample per person), a commercial kit (NAD/NADH Assay Kit, Colorimetric, #ab65348) was used for NAD^+^ detection per manufacturer’s instructions. Briefly, cells were freshly collected (5 million cells/group), quickly washed with 1xPBS (cold), followed by NAD^+^ detection per manufacture’s protocol. All the procedures were performed on ice or at 4 °C to minimize NAD^+^ metabolism.
- For NAD^+^ and its-related metabolites within cells, samples of three biological repeats from primary human cell culture were used for the experiments (Fig. 2 and Supplementary Fig. 3). Experiments were performed using liquid chromatography-mass spectrometry (LC-MS)^33,88^ at Dr. Charles Brenner´s lab (Detailed below).
- For extracellular metabolites, cell culture media were collected 24 h after primary human cell culture (three biological repeats, with data in Supplementary Fig. 4). Samples were then subjected LC-MS (including Label-free QqQ metabolomics and post-processing and bioinformatic analysis) at Dr. Costas A. Lyssiotis´s lab (Detailed below).

### NAD^+^ and its related metabolites within cells

Liquid chromatography-mass spectrometry (LC-MS)^33,88^ was used. To detect intracellular NAD^+^ metabolites (including NMN, NAD^+^, NAAD, ADPR, MeNAM, NA, Inosine, IMP, NADP, cytidine, AMP, ADP, NAM etc.), cells were treated with NR for 24 h, followed by collection of cells for LC-MS as detailed elsewhere^88^. Internal standards: Stable isotope analogs of nucleotides and nucleoside were grown in a yeast broth with universally labeled ^13^C glucose, resulting in all ribose rings being fully labeled. NAAD is the only analyte of interest not having a labeled analog, ^13^C_10_ NAD was used as its internal standard. For the second analysis a mix of ^18^O-NR, ^18^O-Nam, d_4_-NA, d_3_-MeNam, and d_3_- methyl-4-pyridone-3-carboxamide was used as internal standard. For methodology: Cell pellets were received on dry ice and stored at −80 °C. To extract metabolites both sets of internal standards were added along with 400 µL of hot ethanol/HEPES buffer as described previously, working four samples at a time. Extracted samples were stored on ice until all were complete. Samples were then heated at 55 °C with shaking for 3 min. Samples were centrifuged at 16.1 rcf, 4 °C. The supernatants were transferred to fresh microcentrifuge tubes. Calibrators and QC samples were prepared by adding both internal standards and both sets of calibrators to microcentrifuge tubes, 400 µL of hot ethanol/HEPES buffer was added and solvent for all samples, calibrators, and controls was removed in a vacuum centrifuge. For LCMS, the number of pmol of analytes in the samples were determined in two LCMS analyses, which were performed back to back. Separation and quantitation of analytes were performed with a Waters Acquity LC interfaced with a TQD mass spectrometer (Waters) operated in positive ion multiple reaction monitoring mode. NAM related analytes were analyzed using an acid separation, whereas nucleotides and most nucleosides were analyzed using an alkaline separation, each using a dedicated 2.1 × 100 mm Hypercarb column (Thermo Fisher Scientific) held at 60 °C^89^. The autosampler was held at 8 °C. The source temperature of the mass spectrometer was 150 °C and the desolvation temperature was 350 °C. Cone voltages and Collision energies were optimized for each MRM. The LC conditions for the NAM analysis were: Flow 0.20 mL/min, mobile phase “C” 10 mM ammonium acetate + 0.1% formic acid; mobile phase “D” acetonitrile + 0.1% formic acid. For the NAD run the flow rate was 0.353 mL/min, mobile phase “C” 7.5 mM ammonium acetate + 0.05% ammonium hydroxide; mobile phase “D” acetonitrile + 0.05% ammonium hydroxide. The gradients were:

**Table:** 

NAM			NAD		
Time (min)	% C	% D	Time (min)	% C	% D
0	95	5	0	97	3
1.8-	95	5	1.8-	97	3
11	60	40	14	50	50
11.3-	60	40	14.1	10	90
14.3-	10	90	16.2-	10	90
14.4	95	5	17.1	97	3
20	95	5	22	97	3

### Extracellular metabolites

To detect extracellular metabolites, cells were treated with NR for 24 h followed by collection of cell culture media. Cell culture media from three biological repeats/group was used to run LC-MS (including Label-free QqQ metabolomics and post-processing and bioinformatic analysis). Plain MEM media (incubated that had been in cell culture incubator for 24 h) was used as internal background control. Agilent 1290 UHPLC and 6490 Triple Quadrupole (QqQ) Mass Spectrometer (LC-MS) were used in this study. Agilent MassHunter Optimizer and Workstation Software LC/MS Data Acquisition for 6400 Series Triple Quadrupole B.08.00 was used for standard optimization and data acquisition. Agilent MassHunter Workstation Software Quantitative Analysis Version B.0700 for QqQ was used for data analysis. For reversed-phase chromatography (RPC), a Waters Acquity UPLC BEH TSS C18 column (2.1 × 100 mm, 1.7 μm) was used with mobile phase (A) consisting of 0.5 mM NH4F and 0.1% formic acid in water; mobile phase (B) consisting of 0.1% formic acid in acetonitrile. Gradient program: mobile phase (B) was held at 1% for 1.5 min, increased to 80% in 15 min, then to 99% in 17 min and held for 2 min before going to initial condition and held for 10 min. For hydrophilic interaction chromatography (HILIC), a Waters Acquity UPLC BEH amide column (2.1 × 100 mm, 1.7 μm) was used with mobile phase (A) consisting of 20 mM ammonium acetate, pH 9.6 in water; mobile phase (B) consisting of acetonitrile. Gradient program: mobile phase (B) was held at 85% for 1 min, decreased to 65% in 12 min, then to 40% in 15 min and held for 5 min before going to initial condition and held for 10 min. Both columns were at 40 °C and 3 μl of each sample was injected into the LC-MS with a flow rate of 0.2 ml/min. Calibration of TOF MS was achieved through Agilent ESI-Low Concentration Tuning Mix. Optimization was performed on the 6490 QqQ in the positive or negative mode for the RPC or HILIC respectively for each of 220 standard compounds to get the best fragment ion and other MS parameters for each standard. Retention time for each of 220 standards was measured from a pure standard solution or a mix standard solution. The LC-MS/MS method was created with dynamic MRMs with RTs, RT windows and MRMs of all 220 standard compounds. Key parameters of AJS ESI in both the positive and the negative acquisition modes are: Gas temp 275 °C, Gas Flow 14 l/min, Nebulizer at 20 psi, SheathGasHeater 250 °C, SheathGasFlow 11 l/min, and Capillary 3000 V. For MS: Delta EMV 200 V or 350 V for the positive or negative acquisition mode respectively and Cycle Time 500 ms and Cell Acc 4 V for both modes. The QqQ data was pre-processed with Agilent MassHunter Workstation Software Quantitative Analysis and post-processed for further quality control in the programming language R. We calculated coefficient of variation (CV) across replicate samples for each metabolite given a cut-off value of peak areas in both the positive and the negative modes. We then compared distributions of CVs for the whole dataset for a set of peak area cut-off values of 0, 1000, 5000, 10,000, 15,000, 20,000, 25,000, and 30,000 in each mode. A noise cut-off value of peak areas in each mode was chosen by manual inspection of the CV distributions: 5000 for the positive mode and 5000 for the negative mode. Each sample is then normalized by the total intensity of all metabolites to reflect the same protein content as a normalization factor. We then retained only those metabolites with at least 2 replicate 6 measurements. The remaining missing value in each condition for each metabolite was filled with the mean value of the other replicate measurements. Finally, the abundance of each metabolite in each sample was divided by the median of all abundance levels across all samples for proper comparisons, statistical analyses, and visualizations among metabolites. The statistical significance test was done by a two-tailed t-test with a significance threshold level of 0.1. The *p*-values were not adjusted in favor of subsequent manual inspection and more flexible biological interpretation. Those metabolites with *p*-value < 0.1 and CV < 1 were defined to be differential metabolites. Pathway analysis of differential metabolites was done using the webtool of MetaboAnalyst with default settings (metaboanalyst.ca). All other bioinformatics analyses including graphs and plots were also done using R/Bioconductor.

### Whole worm body proteomics

For the whole worm body proteomics, samples of four biological repeats were collected followed by data generation and quantification using methods reported previously^21^. Both control and drug treated worms were cultured in the presence of 5-Fluoro-deoxyuridine (5-FdUrd, 50 µM) to prevent development of progeny in order to produce a synchronized population. L4 stage N2 and *wrn-1* worms were treated with NR (1 mM) for up to 7 days. Subsequently, protein extractions (with extraction buffer 40 mM Tris-HCl pH7.4, 150 mM NaCl, 0.01%, NP-40, 2 mM EDTA; plus protease inhibitors) were performed from four independent experiments. Protein concentration is perfomred with Xpose according to manufactures instructions. Protein extracts (25 μg) were precipitated with 400 μl of ice-cold acetone at −20 °C. After overnight precipitation, samples were centrifuged at 16,000 g for 30 min at 4 °C and the supernatant was discarded. Proteins were re-dissolved in 50 μL of 6 M urea in 50 mM ammonium bicarbonate, pH 7.8. Subsequently, 2.5 μL of 200 mM DTT (Sigma-Aldrich, Norway) was added and the samples were incubated for 30 min at 30 °C to reduce the disulfide bridges. Thiols were then alkylated with 7.5 μL of 200 mM iodoacetamide (IAA Sigma-Aldrich, Oslo, Norway) (1 h, 30 °C, dark), following which the excess of IAA was quenched with 10 μL of 200 mM DTT (30 min, 30 °C). After reducing the urea concentration down to 1 M, proteins were digested with sequencing grade modified trypsin (Promega, Madison, WI, USA) for 1 h at 37 °C, followed by 15 h incubation at 30 °C. The digestion was terminated by adding 5 μL of 50% formic acid and the generated peptides were purified using a Strata C18-E SPE column (Phenomenex, Værløse, Denmark), and dried using a Speed Vac concentrator (Eppendorf, Hamburg, Germany). Tryptic digests from four replicates of each sample were analyzed using an Ultimate 3000 nano-UHPLC system (Dionex, Sunnyvale, CA, USA) connected to a Q Exactive mass spectrometer (ThermoElectron, Bremen, Germany) equipped with a nano electrospray ion source. For liquid chromatography separation, an Acclaim PepMap 100 column (C18, 3 μm beads, 100 Å pore size, 75 μm inner diameter, 50 cm length) was used. A flow rate of 300 nL/min was employed with a solvent gradient of 4–35% B in 207 min, to 50% B in 20 min and then to 80% B in 2 min. Solvent A was aqueous 0.1% formic acid and solvent B contained 0.1% formic acid in 90% acetonitrile. The mass spectrometer was operated in the datadependent mode to automatically switch between MS and MS/MS acquisition. Survey full scan MS spectra (from m/z 300 to 2000) were acquired in the Orbitrap with resolution *R* = 70,000 at m/z 200, after accumulation to a target of 1,000,000 ions in the C-trap. The maximum allowed ion accumulation times were 100 ms. The method used allowed sequential isolation of up to the ten most intense ions, depending on signal intensity (intensity threshold 1.7e4), for fragmentation using higher collision induced dissociation (HCD) at a target value of 1e5 charges and a resolution *R* = 17,500. Target ions already selected for MS/MS were dynamically excluded for 60 s. The isolation window was 2 *m/z* units without offset. The maximum allowed ion accumulation for the MS/MS spectrum was 60 ms. For accurate mass measurements, the lock mass option was enabled in MS mode and the polydimethylcyclosiloxane ions generated in the electrospray process from ambient air were used for internal recalibration during the analysis (with protocol from Fang et al.^21^).

For data analysis, raw data were acquired using Xcalibur v2.5.5 and raw files were processed to generate peak list in Mascot generic format (.mgf) using ProteoWizard release version 3.0.331. Database searches were performed using Mascot in-house version 2.4.0 to search the SwissProt database (Caenorhabditis elegans, 19.05.2015, 26156 proteins) assuming the digestion enzyme trypsin, at maximum one missed cleavage site, fragment ion mass tolerance of 0.02 Da, parent ion tolerance of 10 ppm and oxidation of methionine, acetylation of the protein Nterminus as variable modifications and carbamidomethylation of cysteine as fixed modification. Scaffold (version Scaffold_4.3.4, Proteome Software Inc., Portland, OR) was used to validate MS/MS-based peptide and protein identifications. Peptide identifications were accepted if they could be established at >95.0% probability by the Scaffold Local FDR algorithm. Protein identifications were accepted if they could be established at >99.0% probability.

### Real-time PCR

Cells were treated with/without NR (1 mM) for 24 h. Total RNA was extracted with TriZol (Invitrogen, Carlsbad, CA, USA) reagent, reverse-transcribed using iScript cDNA Synthesis Kit (BIO-RAD, Hercules, CA, USA). mRNA levels were quantified by real-time PCR using a SYBR Green quantitative PCR kit (Thermo Fisher Scientific, Waltham, MA USA) on the MyiQ iCycler real-time PCR detection system (BIO-RAD, Hercules, CA, USA), and then normalized to GAPDH using the 2−ΔΔCT calculation method^90^. Primer sequences used in this study are as follows: NMNAT1 forward, 5′-TCTCCTTGCTTGTGGTTCATTC-3′ and reverse, 5′-TGACAACTGTGTACCTTCCTGT-3′; GAPDH forward, 5′-GAGTCAACGGATTTGGTCGT-3′ and reverse, 5′-GACAAGCTTCCCGTTCTCAG-3′^91^. For the real-time PCR in worms, we collected 50 worms/group and performed PCR using samples from three biological replicates. Primers used for the PCR were detailed elsewhere^10,37^. Values are the means of at least three independent experiments, and standard deviations are indicated as error bars.

### Western blots

Western blotting was used to examine protein expression following methods detailed previously^21^. Briefly, primary human cells (HT01, WRN-Kd, WS01 cells) were collected and prepared using 1x RIPA buffer (Cell Signaling, #9806 S) containing protease inhibitors (Bimake, #B14002) and phosphatase inhibitors (Bimake, #B15002). Proteins were separated on 4–12% Bis-Tris gel (ThermoFisher Scientific, #NP0336BOX) and probed with antibodies. Chemiluminescence detection was performed using a ChemiDoc XRS System. Antibodies used were: β-actin (Santa Cruz, #sc-1616), WRN (Santa Cruz, # sc-5629), CD38 (Santa Cruz, # sc-374650), CD157 (R&D systems, #AF4736), CD73 (R&D systems, #AF5795), PAR (TREVIGEN, #4336-BPC-100), PARP1 (Cell signaling, #9542), AMPK (Cell signaling, #5831), pAMPK (Thr172) (Cell signaling, #2535), pULK1 (Ser555) (Cell signaling, #5869), ULK1 (Cell signaling, #6439), p62 (Cell signaling, #39749), Bcl2L13 (ThermoFisher, # PA5–15043), LC3 (Novus, #NB100–2220), PSD95 (Cell signaling, #3450). All other antibodies were obtained from Cell signaling. Gamma adjustment was used to reduce dark background when necessary. Quantification was performed using ImageJ. Unless elsewhere stated, all the 1st antibodies were with 1000× dilution, while 2nd antibodies were with 10,000× dilution. The original western blots were in the Source Data file with molecular markers labeled.

### Data collection and statistical analysis

Double-blinded methods were used in the *C. elegans* studies (including lifespan, healthspan) and experiments requiring imaging. We used two-tailed unpaired *t*-test for comparison between two groups, or One-Way ANOVA or Two-way ANOVA (followed by Tukey’s test) for comparison among multiple groups. All data were presented as mean ± S.E.M. as indicated with *p value <0.05 considered statistically significant. For lifespan studies, *p* values were derived from log-rank calculations.

### Reporting summary

Further information on research design is available in the Nature Research Reporting Summary linked to this article.

## Supplementary information

Supplementary Information

Description of Additional Supplementary Files

Supplemantary Data 1

Supplementary Data 2

Supplementary Data 3

Reporting Summary

Source Data

## Data Availability

The data that support the findings of this study are available from the corresponding Authors upon reasonable request. The source data underlying Figs. 2i and 5k and Supplementary Figs. 3k, l, m and 8g are provided as a Source Data file. The microarray GEO accession number for the data reported in this paper is GSE108968. The mass spectrometry proteomics data have been deposited to the ProteomeXchange Consortium via the PRIDEpartner repository with the dataset identifier PXD015644. For metabolomic data, the MetaboLights accession codes are MTBLS1221 and MTBLS1223.
