# Supplementary material for: NAD+ augmentation restores mitophagy and limits accelerated aging in Werner syndrome
Source: Nat Commun. 2019 Nov 21;10:5284. doi: 10.1038/s41467-019-13172-8 (PMC6872719; doi:10.1038/s41467-019-13172-8)
Supplement: Supplementary file 1 — Supplementary Information [file 41467_2019_13172_MOESM1_ESM.pdf]

NAD^+ augmentation restores mitophagy and limits accelerated aging in Werner syndrome

Evandro F. Fang et al,. Nature Communications

Supplementary Table 1. Detailed information of primary fibroblasts used in this study

Samples	Age	Sex	Progeroid changes	Cataract	Metabolism	Notes
**Primary fibroblasts**						
WS01	30s	F	pigmented and atrophic skin	Yes	hyperlipidemia type V	From Coriell institute #AG03141 2476C>T
Healthy01 (HT01)	30s	F	no	no	no	From Coriell institute #AG09599
WS02	50s	F	Gray hair, 20s Bird-like face	bilateral, 36yo	Abnormal glucose diabetes 58yo	Diagnosed 57 years old; c.[1105 C>T];[1105C>T]
WS03	50s	F	Gray hair Bird-like face	bilateral, n.a	n.a.	Diagnosed 50 years old; c.[3139-1 G>C];[3139-1 G>C]
WS04	50s	M	Gray hair, 20s Bird-like face	bilateral, 30s	Abnormal glucose; Diabetes, 46 yo dyslipidemia, 46 yo.	Diagnosed 52 years old; c. [1105 C>T];[3139-1 G>C]; Amputation, right leg 50 yo;
WS05	40s	M	Gray hair, 10s Bird-like face	bilateral, 33yo	Abnormal glucose; Diabetes (30s) dyslipidemia (age n.a.)	c.[3139-1 G>C];[3139-1 G>C]
Healthy02	30s	F	no	no	no	
Healthy03	30s	F	no	no	no	
Healthy04	30s	F	no	no	no	
Healthy05	20s	M	no	no	no	

Supplementary Table 2. Detailed information of blood samples used in this study

Samples	Age	Sex	Progeroid changes	Cataract	Metabolism	Notes
**Blood samples**						
WS02	50s	F	Gray hair, 20s<br>Bird-like face	bilateral, 36yo	Abnormal glucose<br>diabetes 58yo	Diagnosed 57 years old;<br>c.[1105 C>T];[1105C>T]
WS03	50s	F	Gray hair<br>Bird-like face	bilateral, n.a	n.a.	Diagnosed 50 years old;<br>c.[3139-1 G>C];[3139-1 G>C]
WS04	50s	M	Gray hair, 20s<br>Bird-like face	bilateral, 30s	Abnormal glucose;<br>Diabetes, 46 yo<br>dyslipidemia, 46 yo.	Diagnosed 52 years old;<br>c. [1105 C>T];[3139-1 G>C];<br>Amputation, right leg 50 yo;
WS05	40s	M	Gray hair, 10s<br>Bird-like face	bilateral, 33yo	Abnormal glucose;<br>Diabetes (30s)<br>dyslipidemia (age n.a.)	c.[3139-1 G>C];[3139-1 G>C]
WS06	30s	M	-	bilateral, n.a	Diabetes	c.[3139-1G>C];[1720+1 G>A]
WS07	40s	M	Skin atrophy<br>Bird-like face	bilateral, n.a	No diabetes	c.[3139-1 G>C];[3139-1 G>C]
WS08	30s	F	Gray hair<br>Muscle atrophy<br>Skin atrophy	bilateral, n.a	No diabetes	c.[3139-1 G>C];[3139-1 G>C]
WS09	30s	M	Skin atrophy<br>Bird-like face	bilateral, n.a	Pre-diabetes<br>dyslipidemia	c.[3139-1 G>C];[2959 C>T]
WS10	30s	F	Gray hair/alopecia	bilateral, n.a	No diabetes	c.[3139-1 G>C];[3139-1 G>C]
WS11	40s	M	Skin atrophy<br>Bird-like face	bilateral, n.a	Diabetes	c.[3139-1 G>C];[3139-1 G>C]
Healthy02	20s	M	no	no	no	
Healthy03	30s	F	no	no	no	
Healthy04	30s	F	no	no	no	
Healthy05	30s	F	no	no	no	
Healthy06	20s	M	no	no	no	
Healthy07	30s	M	no	no	no	
Healthy08	30s	F	no	no	no	
Healthy09	40s	M	no	no	no	
Healthy10	20s	F	no	no	no	
Healthy11	40s	M	no	no	no	
Healthy12	30s	M	no	no	no	
Healthy13	30s	M	no	no	no	

*Whole blood samples, others were plasma samples
n.a., not available

Supplementary Table 3. List of significantly changed fat metabolites analyzed in N2 and *wrn-1* with/without NR treatment.

	wrn-1(veh.)/N2(veh.)	p-value (t-test)	N2(NR)/N2(veh.)	p-value (t-test)	wrn-1(NR)/wrn-1(veh.	p-value (t-test)
acs-5	0.369725641	0.040801757	1.364919224	0.298075323	1.70108453	0.423813411
IBP-2	1.189700177	0.026777991	1.129484772	0.161602938	1.13060255	0.014288363
ACS-11	0.730307793	0.017548717	0.573983313	0.009286298	0.827217362	0.236828829
ACS-13	1.365918998	0.83602773	0	0.355917684	0	0.355917684
ECH-1,2	1.069539339	0.138931468	0.895644048	0.004148639	1.086938241	0.15437051
ACLY-2	1.156324892	0.107767707	1.019260351	0.787601324	0.740656679	0.025065597
CPT-1	1.083448864	0.623830894	0.831861405	0.616636656	1.834958518	0.021048703
CPT-2	0	3.08379E-05	0.202060656	0.011229575		
ACDH-3	1.144291455	0.035501946	1.008840995	0.8991175	0.961617669	0.548547638
ACDH-9	1.006567568	0.894441401	1.08205561	0.407319144	1.220602572	0.002729574
ACDH-12	0.652708919	0.011546844	0.9153004	0.477695161	1.475501741	0.0267301
FASN-1	0.912505733	0.286407825	0.804920238	0.003303639	0.890775274	0.21942286
CTS-1	0.996714062	0.967234606	1.17408593	0.038783797	1.07071481	0.348026333
SDHA-1	0.907084452	0.208296203	0.943099663	0.444816196	1.215762358	0.024432209
ACO-2	1.234670874	0.021765087	1.075201873	0.187568398	0.820450604	0.016185538
IDHG-1	0.906553365	0.35207741	0.754686347	0.025179012	1.012248862	0.933242872
LBP-1	1.169467926	0.001719662	0.989812667	0.902342593	1.047067319	0.239151294
LBP-2	1.198358759	0.027213946	1.159047581	0.091083264	1.168776193	0.002215794
cluster GPD-1	1.299841964	0.001460697	0.952418939	0.382751265	0.792771189	0.000418513
GPD-1	1.307624148	0.002333478	0.956772211	0.39408815	0.792510682	0.002011916
GPD-4	1.349226838	0.000307544	0.965702674	0.439503277	0.775637429	0.000118735
GPD-2	1.130828318	0.102186086	1.12956579	0.043912304	1.004311984	0.957156592
cluster GPD-3	1.1307488	0.065147123	1.127998176	0.03996678	1.00482793	0.950849295
GPD-3	1.137612528	0.048214783	1.128362099	0.028924243	0.986915307	0.851044095
ACS-4	1.300213886	0.004522039	0.854367671	0.005695774	0.738634261	0.015911116
ACS-5	0.369725641	0.040801757	1.364919224	0.298075323	1.70108453	0.423813411
MDH-1	1.207997873	0.011775306	1.129867497	0.149935581	0.964208272	0.232443674
SDHA-1.2	1.316548287	0.011623122	1.082797177	0.229162754	0.774157456	0.024835996
SUCG-1 (C50F7,4 in WormBase)	1.106041467	0.349382277	0.740330208	0.037794143	0.797688496	0.072233187
HXK-2 (H25P06,1 in WormBase)	0.715248821	0.011121329	0.556930801	0.003658246	0.584322602	0.10980754
PFK-1	1.130192053	0.023577949	1.105548584	0.228231167	1.056542224	0.506600469

p-value	
less than 0.001	
between 0.01 and 0.001	
between 0.05 and 0.01	
Fold change:	
	sign. decreased
	sign. increased

Supplementary Table 4. A summary of lifespan values in different conditions

Experiments	Groups	Mean lifespan	Statistics
a	Condition	Mean lifespan ±SEM	p value vs. wrn-1(gk99) (Veh)
	N2 (Veh)	17±0.50	<0.001
	wrn-1(gk99) (Veh)	14±0.50	-
	wrn-1(gk99) (NR)	18±0.44	<0.001
	wrn-1(gk99);dct-1(RNAi) (Veh)	14±0.38	>0.05
	wrn-1(gk99);dct-1(RNAi) (NR)	15±0.38	<0.05<br><0.05 to *wrn 1(gk99);dct-1(RNAi) (Veh)*
b	Condition	Mean lifespan (Mean ± SEM)	P-Value to *wrn-1* veh
	N2 veh	20.6 ± 0.25	
	N2 NR	22.0 ± 0.23	<0.001
	N2 NMN	22.4 ± 0.25	<0.001
	wrn-1 veh	13.9 ± 0.43	<0.001
	wrn-1 NR	18.1 ± 0.48	<0.001
	wrn-1 NMN	19.8 ± 0.45	<0.001
	wrn-1 SRT1720	16.5 ± 0.46	<0.001
	wrn-1 Ola	15.0 ± 0.44	<0.05
c	Condition	Mean lifespan (Mean ± SEM)	p-value to *wrn-1* (veh)
	N2 veh	19.0 ± 0.5	<0.01
	wrn-1 veh	16.8 ± 0.2	-
	wrn-1 NR	20.3 ± 0.5	<0.001
	wrn-1 NMN	21.3 ± 0.4	<0.001
	wrn-1 NAD^+	20.8 ± 0.4	<0.001
	wrn-1 NAM	17.9 ± 0.7	>0.05
d	Condition (Starting point of treatment)	Mean lifespan (Mean ± SEM, Days)	p-value to N2 veh
	N2 veh	21.0 ± 0.53	-
	N2 NR (egg)	22.4 ± 0.64	0.012
	wrn-1 veh	15.0 ± 0.46	< 0.0001
e	Condition	Mean lifespan (Mean ± SEM, Days)	p-value to *wrn-1* veh.
	wrn-1 veh	15.0 ± 0.46	-
	wrn-1 NR (egg)	18.0 ± 0.61	0.0007
	wrn-1 NR (L4)	17.3 ± 0.57	< 0.0001
	wrn-1 NR (D3)	18.3 ± 0.70	< 0.0001
	wrn-1 NR (D5)	16.3 ± 0.70	0.040
f	Condition	Mean lifespan ±SEM	p-value to *wrn-1* Veh
	N2 Veh	17±0.57	<0.0001
	wrn-1 Veh	10±0.56	
	wrn-1 NR	16±0.70	<0.0001
	wrn-1 UA	12±0.50	<0.05

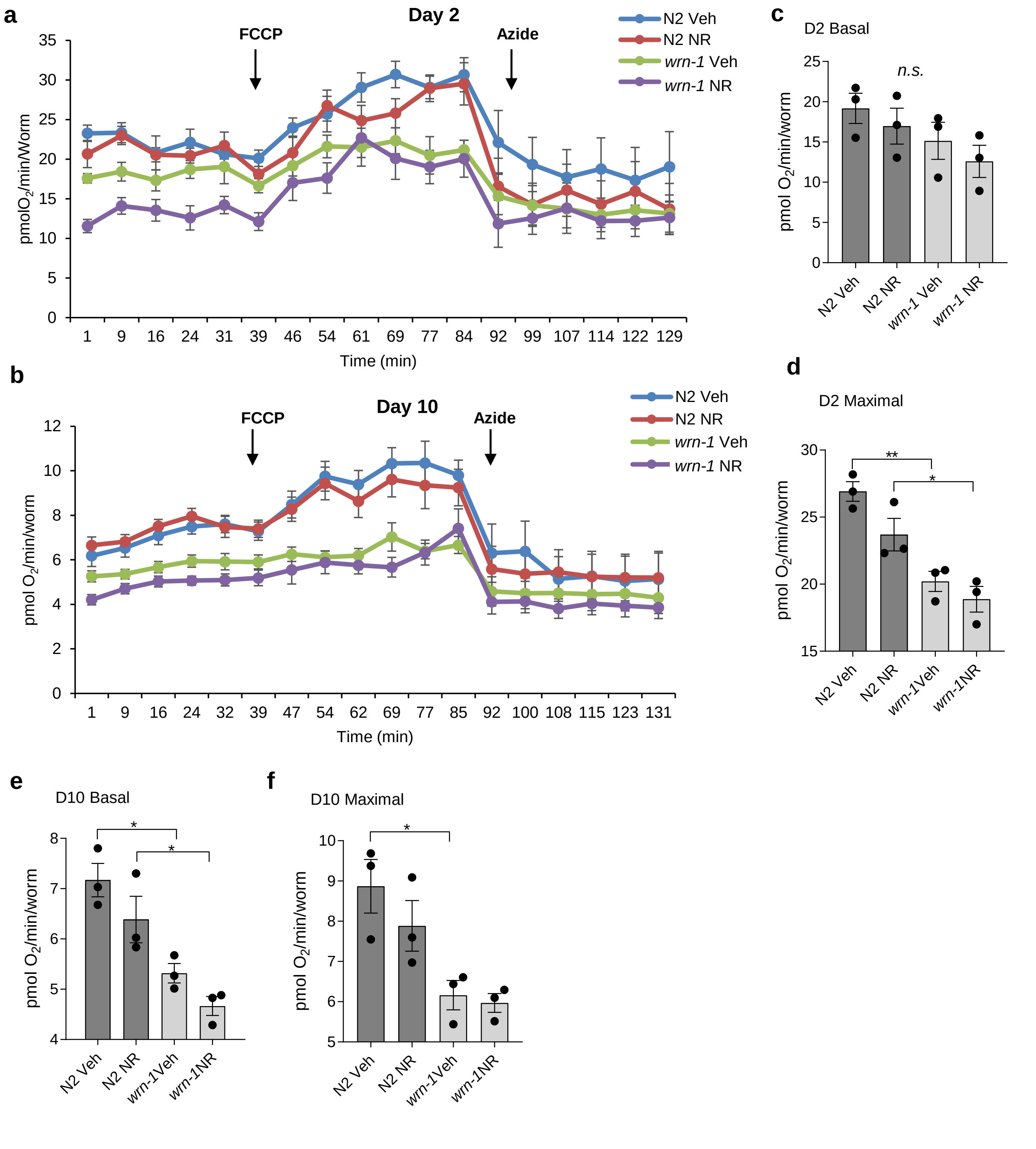

Supplementary Fig. 1. Effect of NR treatment on the oxygen consumption rate in young and old *wrn-1* worms.

Wild type N2 and *wrn-1*(*gk99*) worms were exposed with/without NR (1 mM) from L4 stage until experiment. On adult day 2 or adult day 10, oxygen consumption rates were examined using the XFe96. All data are shown in mean ± S.E.M (n = 3 biologically independent experiments).

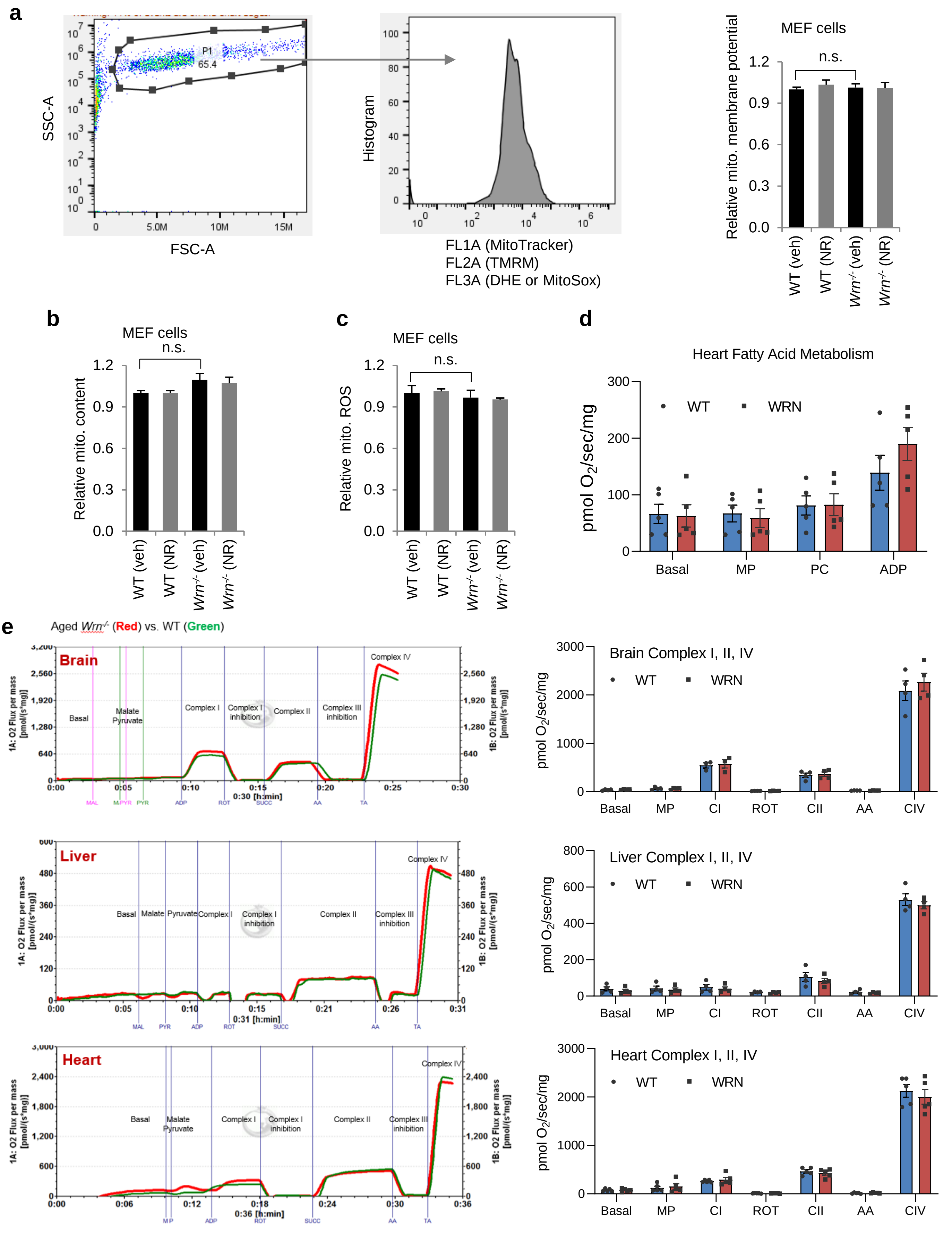

Supplementary Figure 2

Supplementary Fig. 2. Changes of mitochondrial function in mouse and *C. elegans* models of WS.

(a-c) Flow cytometry analysis of relative values of mitochondrial membrane potential (a), mitochondrial content (b), and mitochondrial ROS (c) in WT and $Wrn^{-/-}$ MEFs treated with/without NR (1 mM, 24 h). For the left two panels of (a), a sorting strategy was presented with the P1 gating panel was used for all the data panels. For the MitoTracker staining, FL1A was used; For the TMRM staining, the FL2A was used; For DHE or MitoSox staining, the FL3A was used. All data are shown in mean ± S.E.M (n = 3 biologically independent experiments). n.s., $p>0.05$.

(d) No changes of fatty acid metabolism in the mitochondria extraction from heart tissues between the WT and the $Wrn^{-/-}$ mice. Data are shown in mean ± S.E.M. (n = 5 mice).

(e) Oroboros analysis showing no difference of mitochondrial function in the brain, liver, or heart tissues between aged $Wrn^{-/-}$ mice (15-17.5 months) and WT littermates. (n = 4~5 mice).

For c-f, Two-way ANOVA followed by Tukey's post hoc tests: n.s., $p>0.05$ *, $p<0.05$, **, $p<0.01$, ***, $p<0.001$.

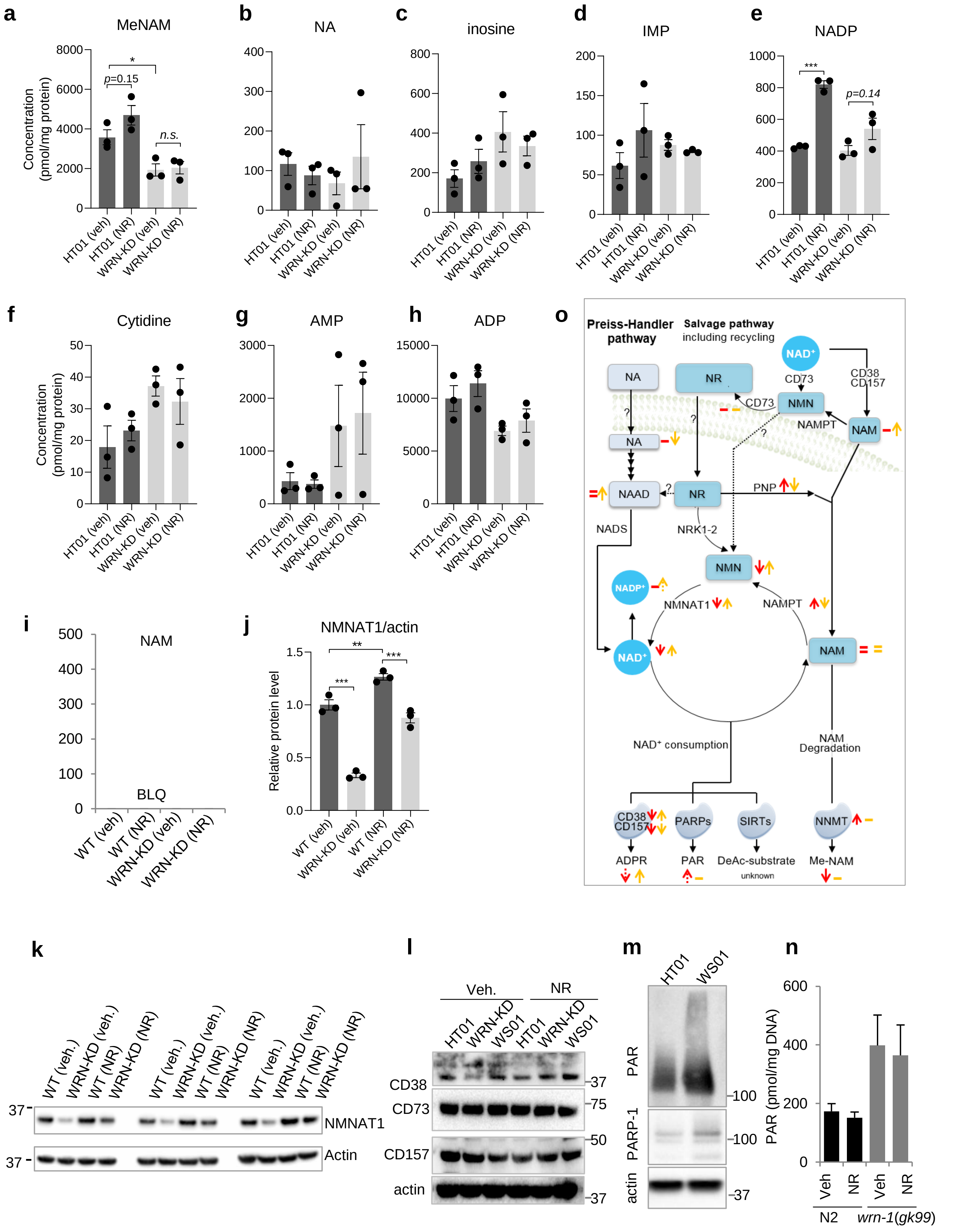

Supplementary Figure 3

Supplementary Fig. 3. Changes of NAD^+ metabolic profiles, NAD^+ metabolism-related enzymes, and mRNA level of *nmnat1* in designated human cells with/without NA treatment.

(a-i) Liquid chromatography-mass spectrometry (LC-MS) data showing changes of methylated nicotinamide (MeNAM), nicotinic acid (NA), inosine, IMP, nicotinamide adenine dinucleotide phosphate (NADP), cytidine, adenosine monophosphate (AMP), adenosine diphosphate (ADP), and nicotinamide (NAM) in HT01 and WRN-KD cells before and after NR treatment (1 mM, 24 h). Data are shown in mean ± S.E.M (n = 3 samples/group; Two-way ANOVA followed by Tukey's post hoc tests: n.s., $p>0.05$)

(j) Quantification of protein expression levels from Fig. 2i. n = 3 biologically independent experiments.

(k) Western blotting showing changes of expression levels of designated proteins. n = 3 biologically independent experiments.

(l) Western blotting showing changes of expression levels of proteins involved in NAD^+ consumption in designated conditions.

(m) Western blotting showing changes of designated proteins in the HT01 and the WS01 cells.

(n) Mass spectrometry data showing changes of PAR levels in the N2 and the *wrn-1(gk99)* worms with/without NR treatment (1 mM from L4 stage).

(o) A summary of the changes of the NAD^+ metabolites as well as NAD^+ synthesis- or consumption-related enzymes. Red, WRN-KD (veh) vs. HT01 (veh); Orange, WRN-KD (NR) vs. WRN-KD (veh); Solid arrows, with difference; dashed arrows, with a trend of difference; one bar, no difference; two bars, undetectable. The summary was based on the data from Fig. 2 and Supplementary Fig. 3, as well other results in this study.

For a-j and n, Two-way ANOVA followed by Tukey's post hoc tests: n.s., $p>0.05$, *, $p<0.05$, **, $p<0.01$, ***, $p<0.001$.

For Supp. Fig. 3k, l, and m, source data are provided as a Source Data file.

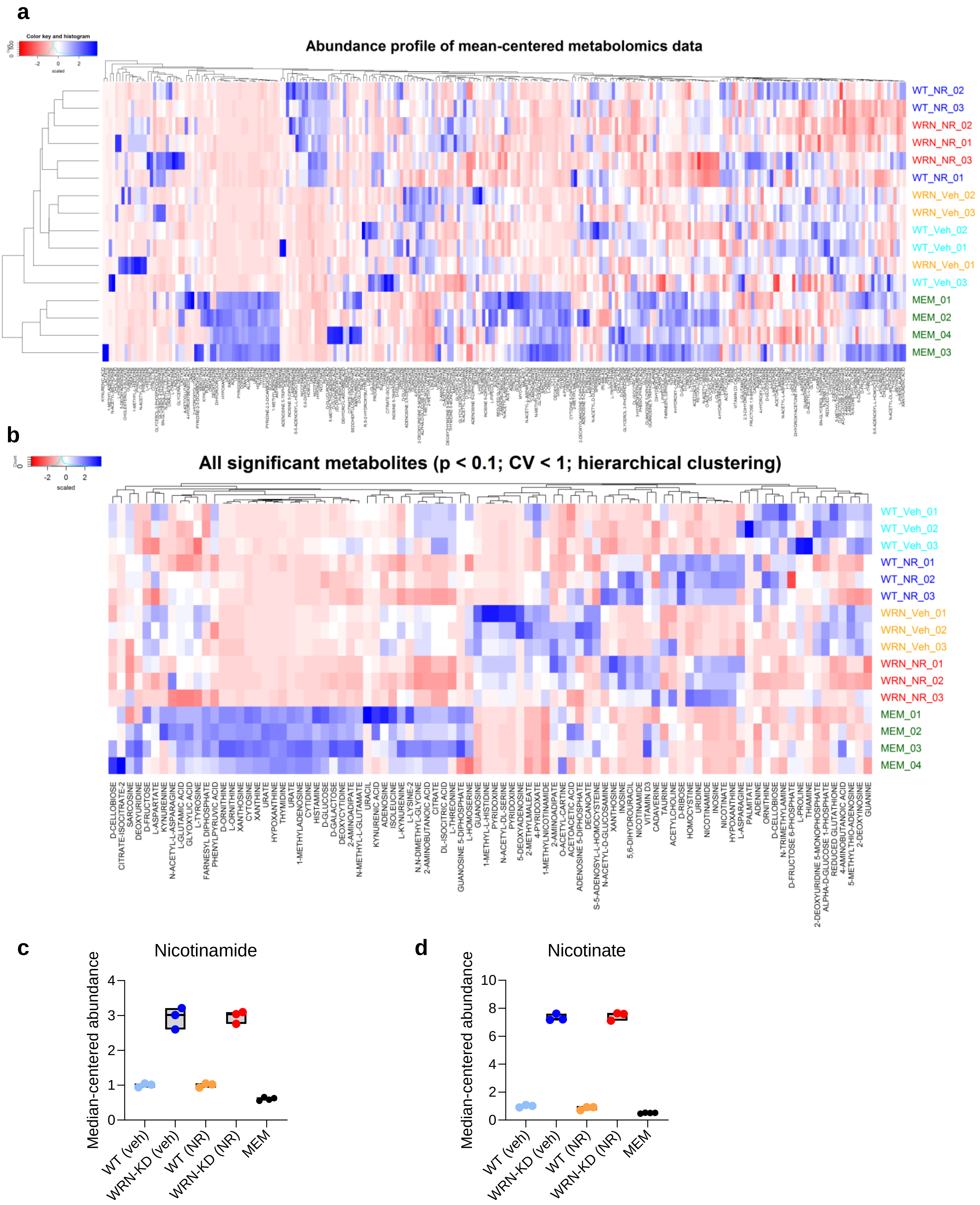

Supplementary Figure 4

Supplementary Fig. 4. Changes of extra-cellular NAD$^+$ metabolites.
Extracellular metabolic profiles were examined for media incubated in the HT01 and the WRN-KD cells treated with NR (1 mM) or Mock. Growth media (MEM media, no FBS, incubated at 37 °C for 24 h) were used as internal background control.
(a) Targeted liquid chromatography-mass spectrometry (LC-MS) based metabolomics data showing all the detected metabolites (n = 3 separate cultures/group).
(b) Data from (A) for those metabolites which exhibited significance (n = 3 separate cultures/group).
(c-d) Relative levels of nicotinamide and nicotinate in different conditions (n = 3 separate cultures/group) (also presented in the heat maps above). Box plot was used for data presentation: coloured box stands for median ± upper quartile/lower quartile, with whisker variations indicating upper hinge and lower hinge.

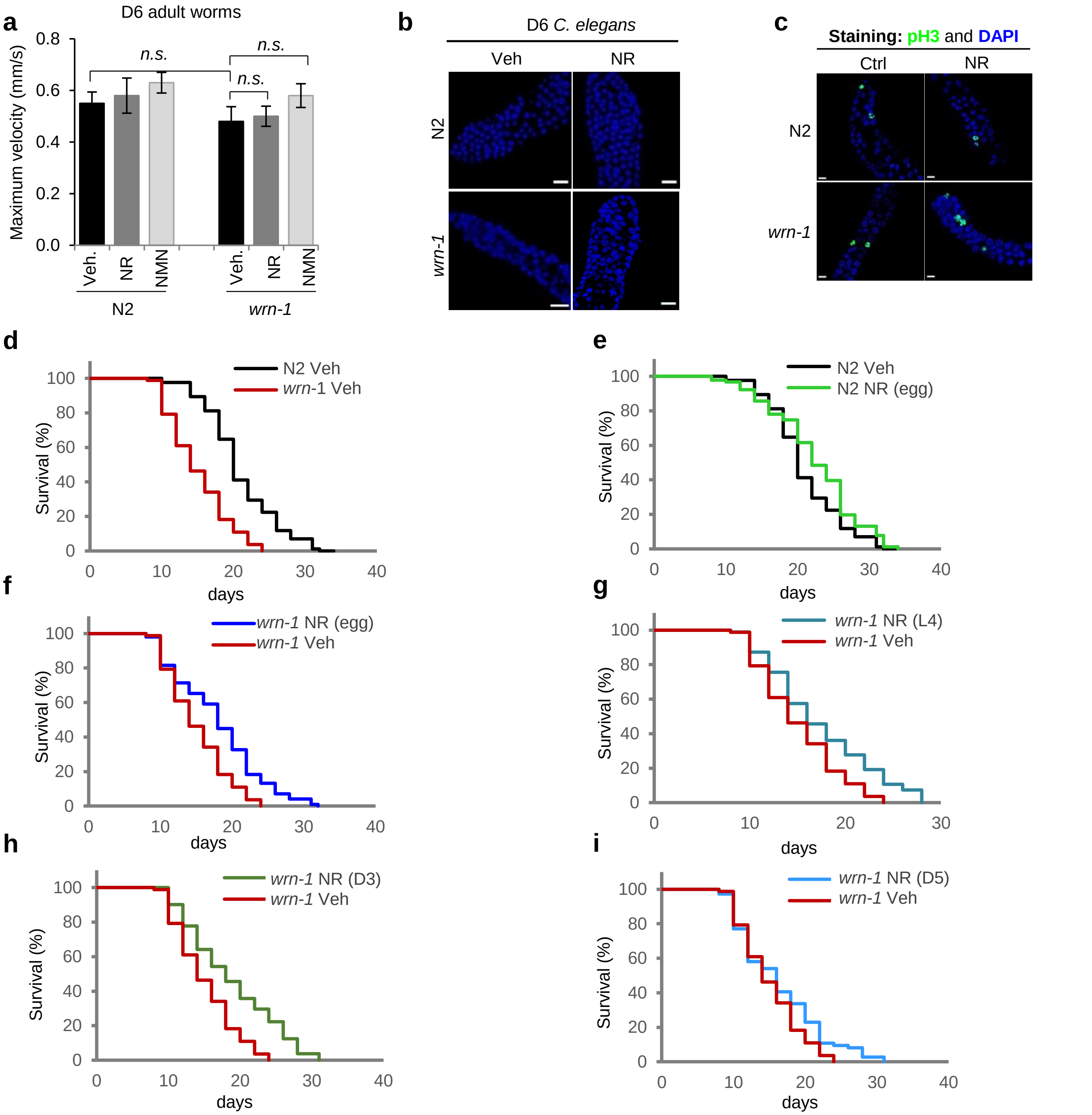

Supplementary Fig. 5. Changes of lifespan in the N2 and *wrn-1* worms with NR exposure at different stages of life or with NAD⁺/different NAD⁺ precursors.

(a) Changes of maximum velocity between adult D6 N2 and the *wrn-1(gk99)* worms in different conditions. Data are shown in mean ± S.E.M (n = 20 worms in each condition).

(b) A representative set of images on the changes of the numbers of germ line-localized mitotic cells in the designated groups of D6 worms. Quantification was in Fig. 3e. Scale bars, 10 μm

(c) A representative set of images on the changes of the numbers of pH3⁺ mitotic cells in the designated groups of D6 worms. Quantification was in Fig. 3f. All the worms were exposed to 90 Gy γ-radiation. Scale bars, 10 μm

(d-i) Effects of NR treatment at different stages of the worm life on the worm lifespan. NR was given at 1 mM at different stages. Quantifications were summarized in Supplementary Table 4.

All the lifespan studies were repeated 2-7 times. Two-way ANOVA followed by Tukey's *post hoc* tests was used for data analysis.

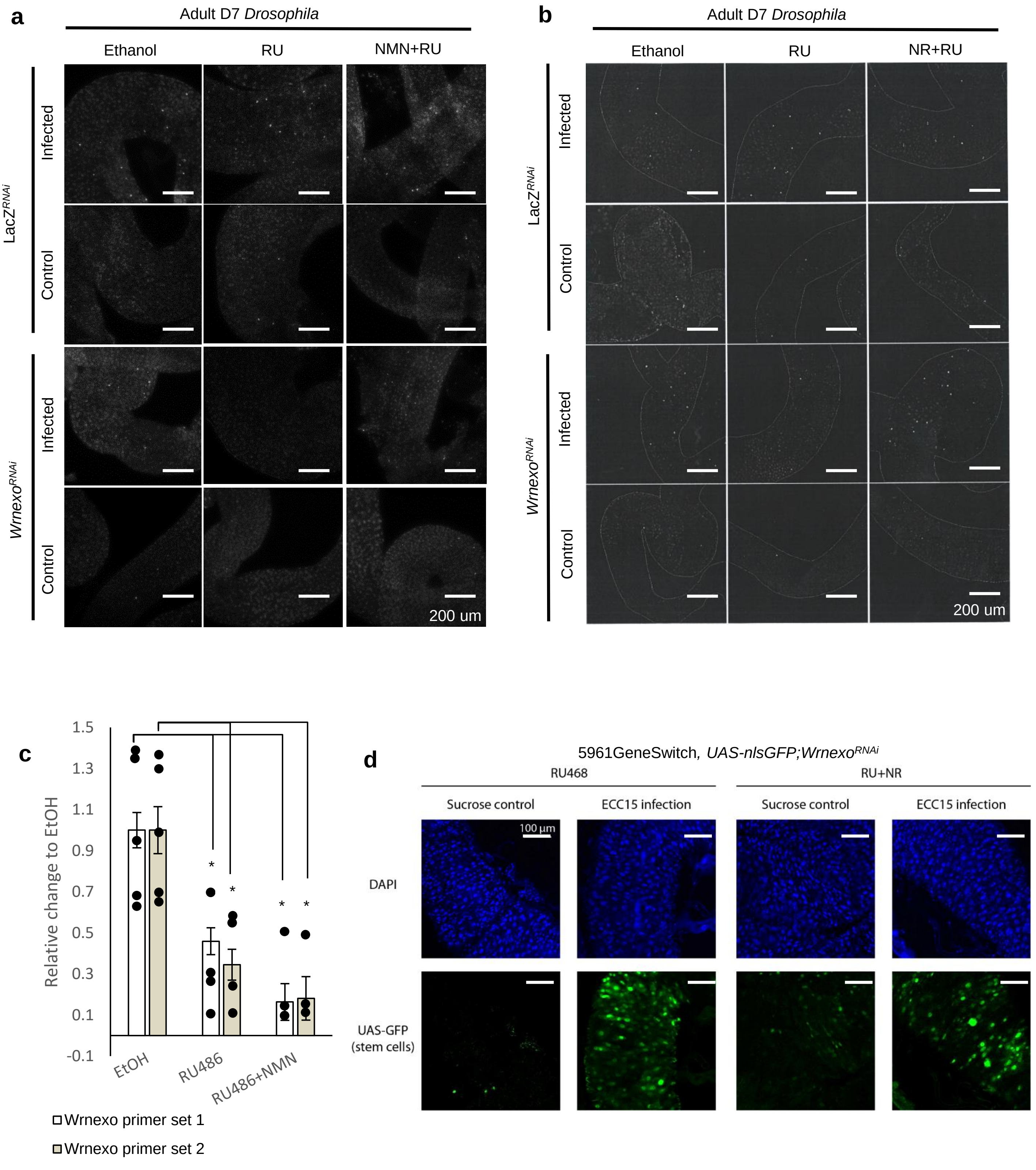

Supplementary Figure 6

Supplementary Fig. 6. NR and NMN improve gut stem cell proliferation in the WS flies.
(a-b) Effect of NMN (a) or NR (b) on average numbers of gut damage-induced proliferating intestinal stem cells (ISCs). The WT and the *Wrnexo*^{RNAi} flies were treated with vehicle, NMN (a) or NR (b) and the total numbers of the pH3^{+} gut stem cells/gut (i.e., dividing stem cells) were counted on adult day 7. The 5961GeneSwitch-Gal4 line was used for the study. This line expresses an inactivated form of Gal4 in intestinal stem cells, which is activated in the presence of the drug RU486. A set of representative images are shown here with quantified data shown in Fig. 3h and Fig. 3i, respectively. Scale bars, 200 µm.
(c) Knockdown efficiency of the WS flies. Confirmation of *Wrnexo* KD in the *Wrnexo*^{RNAi} flies treated with either EtOH (WT), RU486 (*Wrnexo* KD) or RU486 + 5 mM NMN for 7 days. Two different primer sets against *Wrnexo* were used to confirm KD with 4-5 flies/group. *, p <0.05 using One-way ANOVA.
(d) The *UAS-nlsGFP* construct was used to evaluate the total number of intestinal stem cells, to rule out cell death as a factor in the proliferative response. Intestinal stem cells were still present in *Wrnexo*^{RNAi} flies, before and after infection. Also, infection stimulated stem cell numbers. Scale bars, 100 µm.

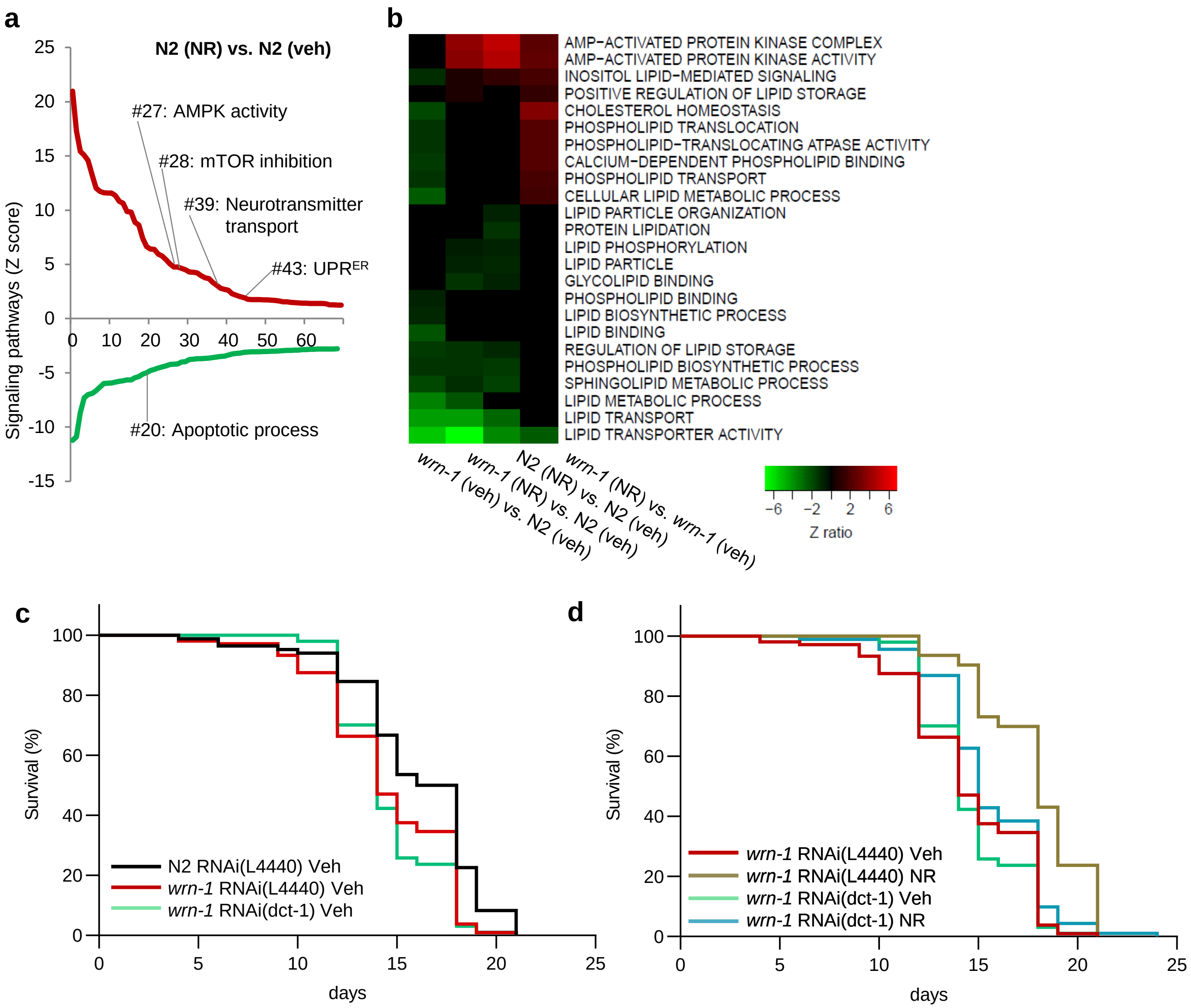

Supplementary Fig. 7. NAD⁺ replenishment normalizes the transcriptomic profiles of WRN.
The *wrn-1(gk99)* and the N2 worms were treated with NR (1 mM) from the L4 stage, followed by changing to fresh drug plate on adult Day4. The worms were collected on adult D7 for transcriptomic analysis.
(a) GO term analysis of transcriptomic analysis revealed pathways related to mitophagy/autophagy, energy expenditure and UPR were among the changed pathways after NR treatment in N2 worms.
(b) Gene-set-enrichment analysis demonstrates upregulated and downregulated signaling pathways (GO pathways) in the D7 N2 and the *wrn-1(gk99)* worms treated with/without NR (1 mM from L4). The GO terms were ranked on the basis of enrichment scores. Heat map data showing changes of the GO terms related to lipid metabolism among 4 different comparisons.
(c) Lifespan curves of vehicle treated N2 RNAi(L4440), *wrn-1* RNAi(L4440) and *wrn-1* RNAi(*dct-1*) worms at 25 °C.
(d) Lifespan curves of the *wrn-1* RNAi(L4440) and *wrn-1* RNAi(*dct-1*) worms with/without NR at 25 °C.

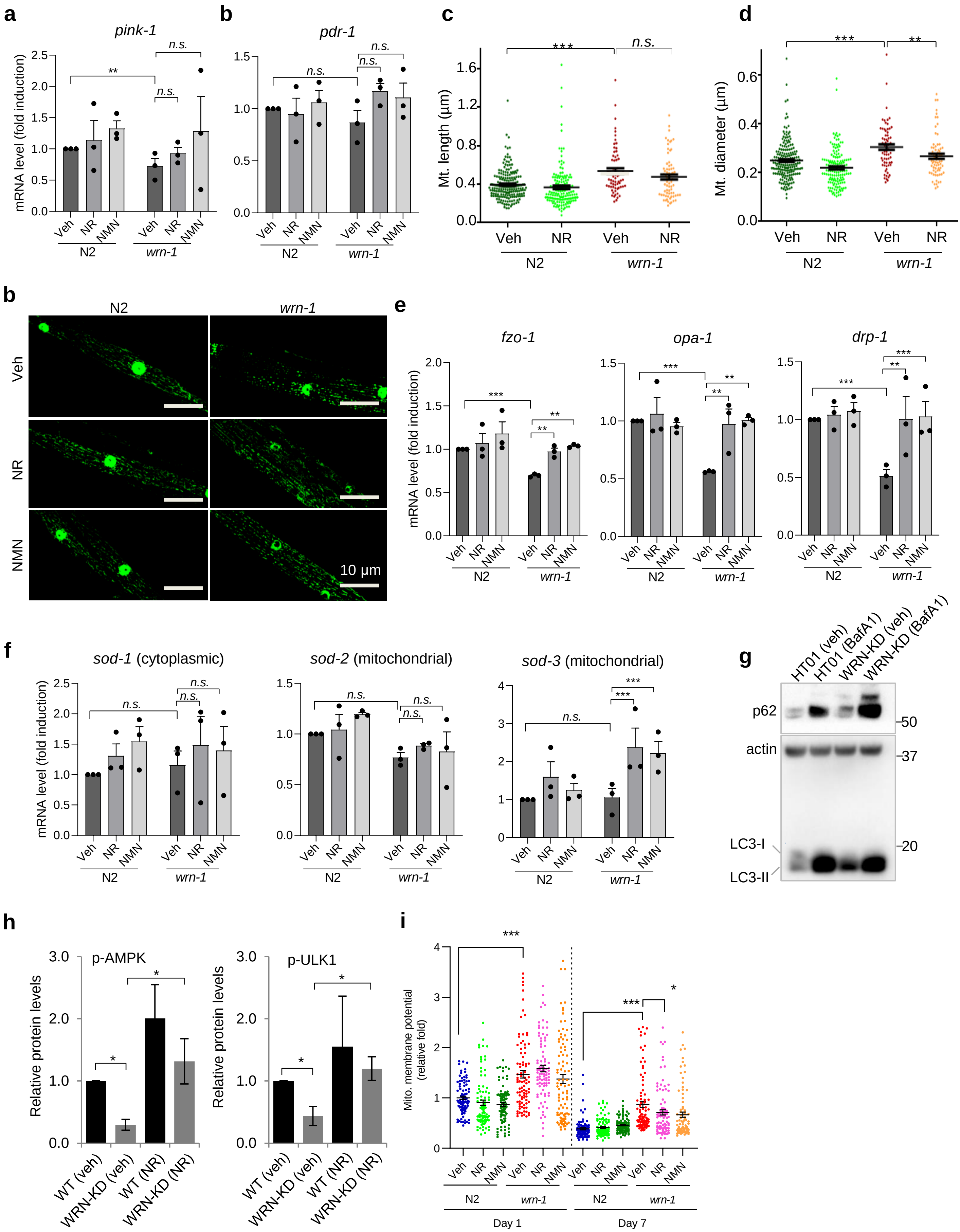

Supplementary Figure 8

Supplementary Fig. 8. Changes of mitophagy and mitochondrial fusion-fission related genes, as well as mitochondrial morphology/function in N2 and the *wrn-1*(*gk99*) worms in different conditions.
(a) The mRNA levels of *pink-1* and *pdr-1* in the NR and NMN-treated N2 and the *wrn-1(gk99)* worms. Data are shown in mean ± S.E.M. (n = 3 biologically independent experiments).
(b) Representative images of muscle mitochondrial morphology of adult D7 N2 and *wrn-1*(*gk99*) worms under different conditions. A *myo-3::gfp* reporter gene was expressed in body wall muscles for imaging. Data shown in mean ± S.E.M. (n = 10-20 worms/condition). Scale bars, 10 µm.
(c-d) Adult D7 worms in different conditions were collected for electron microscopy, and the length as well as diameter of mitochondria in different groups were quantified. Data are shown in mean ± S.E.M. (n = 100 mitochondria in each condition).
(e-f) The mRNA levels of designated in the NR and NMN-treated N2 and the *wrn-1(gk99)* worms. Data are shown in mean ± S.E.M. (n = 3 biologically independent experiments).
(g) Representative western blot data showing higher basal level of macro autophagy in the WRN-KD cells, as well as no defect of autophagic machinery. Bafilomycin A1 (BafA1), a lysosome inhibitor (100 nM, overnight). Source data are provided as a Source Data file.
(h) Quantification of protein expression levels from Fig. 5j.
(i) Comparison of mitochondrial membrane potential (MMP) between the N2 and the *wrn-1(gk99)* worms in ageing and NR or NMN treatment conditions. TMRM was used for quantification of MMP. NR (1 mM), NMN (1 mM) treated from the L4 stage. The data are shown in mean ± S.E.M. with values pooled from three independent biological repeats (n = 90 worms/group).
For a, c-f, h-j, Two-way ANOVA followed by Tukey's post hoc tests: n.s., p>0.05, *, p<0.05, **, p<0.01, ***, p<0.001.

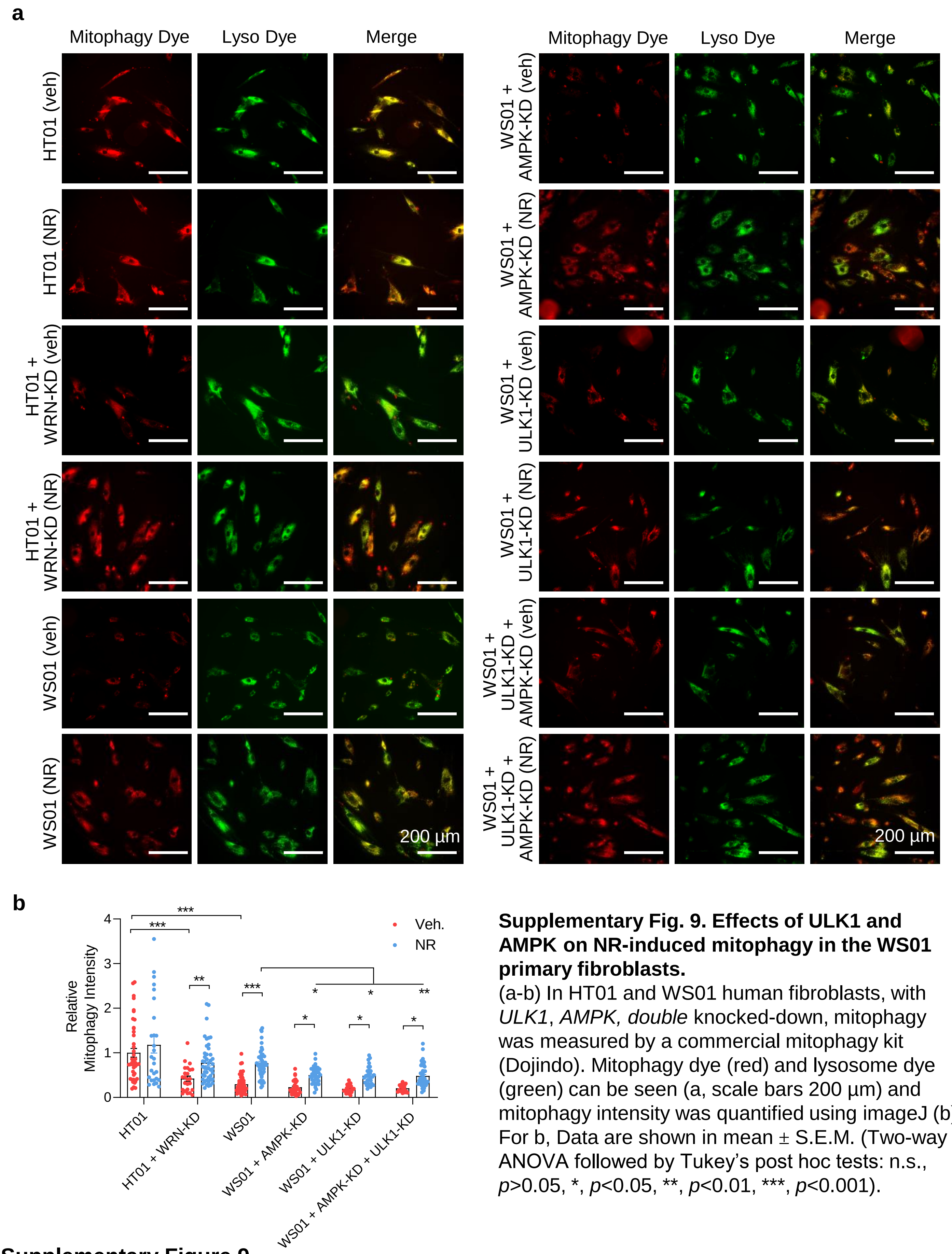

Supplementary Fig. 9. Effects of ULK1 and AMPK on NR-induced mitophagy in the WS01 primary fibroblasts.
(a-b) In HT01 and WS01 human fibroblasts, with *ULK1*, *AMPK*, *double* knocked-down, mitophagy was measured by a commercial mitophagy kit (Dojindo). Mitophagy dye (red) and lysosome dye (green) can be seen (a, scale bars 200 μm) and mitophagy intensity was quantified using imageJ (b). For b, Data are shown in mean ± S.E.M. (Two-way ANOVA followed by Tukey's post hoc tests: n.s., $p>0.05$, *, $p<0.05$, **, $p<0.01$, ***, $p<0.001$).

Supplementary Figure 9
