# Supplementary material for: NAD+ augmentation restores mitophagy and limits accelerated aging in Werner syndrome
Source: Nat Commun. 2019 Nov 21;10:5284. doi: 10.1038/s41467-019-13172-8 (PMC6872719; doi:10.1038/s41467-019-13172-8)
Supplement: Supplementary file 2 — Description of Additional Supplementary Files [file 41467_2019_13172_MOESM2_ESM.docx]

**Description of Additional Supplementary Files**

File Name: Supplementary Data 1

Description: A list of all worm metabolites acquired. Raw data of all the metabolites acquired from the whole body tissues of both N2 and the *wrn-1* worms with different treatments) (this also includes the statistical analysis of the metabolites used for Fig. 6a and b)

File Name: Supplementary Data 2

Description: Mass spectrometry for Fig. 2

File Name: Supplementary Data 3

Description: Mass spectrometry for Supplementary Fig. 4
