# Supplementary material for: NAD+ augmentation restores mitophagy and limits accelerated aging in Werner syndrome
Source: Nat Commun. 2019 Nov 21;10:5284. doi: 10.1038/s41467-019-13172-8 (PMC6872719; doi:10.1038/s41467-019-13172-8)
Supplement: Supplementary file 6 — Reporting Summary [file 41467_2019_13172_MOESM6_ESM.pdf]

nature research

Corresponding author(s): Evandro F. Fang and Vilhelm Bohr

Last updated by author(s): 11 July 2019

Reporting Summary

Nature Research wishes to improve the reproducibility of the work that we publish. This form provides structure for consistency and transparency in reporting. For further information on Nature Research policies, see Authors & Referees and the Editorial Policy Checklist .

Statistics

For all statistical analyses, confirm that the following items are present in the figure legend, table legend, main text, or Methods section.

n/a	Confirmed	
☐	☒	The exact sample size (n) for each experimental group/condition, given as a discrete number and unit of measurement
☐	☒	A statement on whether measurements were taken from distinct samples or whether the same sample was measured repeatedly
☐	☒	The statistical test(s) used AND whether they are one- or two-sided<br>*Only common tests should be described solely by name; describe more complex techniques in the Methods section.*
☐	☒	A description of all covariates tested
☐	☒	A description of any assumptions or corrections, such as tests of normality and adjustment for multiple comparisons
☐	☒	A full description of the statistical parameters including central tendency (e.g. means) or other basic estimates (e.g. regression coefficient) AND variation (e.g. standard deviation) or associated estimates of uncertainty (e.g. confidence intervals)
☒	☐	For null hypothesis testing, the test statistic (e.g. F, t, r) with confidence intervals, effect sizes, degrees of freedom and P value noted<br>*Give P values as exact values whenever suitable.*
☒	☐	For Bayesian analysis, information on the choice of priors and Markov chain Monte Carlo settings
☒	☐	For hierarchical and complex designs, identification of the appropriate level for tests and full reporting of outcomes
☒	☐	Estimates of effect sizes (e.g. Cohen's d, Pearson's r), indicating how they were calculated

Our web collection on statistics for biologists contains articles on many of the points above.

Software and code

Policy information about availability of computer code

Data collection	BD Accuri™ C6 Plus Flow Cytometer (with attached software) was used to collect the flow cytometry data.
Data analysis	Microsoft Excel (2016) and Graphpad Prism 7 software were used for data analysis. ImageJ with the plug in ObjectJ (https://sils.fnwi.uva.nl/bcb/objectj/download/) was used for morphological quantification of mitochondria.

For manuscripts utilizing custom algorithms or software that are central to the research but not yet described in published literature, software must be made available to editors/reviewers. We strongly encourage code deposition in a community repository (e.g. GitHub). See the Nature Research guidelines for submitting code & software for further information.

Data

Policy information about availability of data

All manuscripts must include a data availability statement. This statement should provide the following information, where applicable:
- Accession codes, unique identifiers, or web links for publicly available datasets
- A list of figures that have associated raw data
- A description of any restrictions on data availability

The data that support the findings of this study are available from the corresponding authors upon reasonable request. The source data underlying Figs 2i and 5k and Supplementary Figs 3k, 3l, 3m and 8g are provided as a Source Data file. The microarray GEO accession number for the data reported in this paper is GSE108968.

Field-specific reporting

Please select the one below that is the best fit for your research. If you are not sure, read the appropriate sections before making your selection.

- [x] Life sciences
- [ ] Behavioural & social sciences
- [ ] Ecological, evolutionary & environmental sciences

For a reference copy of the document with all sections, see nature.com/documents/nr-reporting-summary-flat.pdf

Life sciences study design

All studies must disclose on these points even when the disclosure is negative.

Sample size	Sample sizes for behavioral experiments were determined by the current standard used for mice in behavioral neuroscience experiments, based on the minimal amount of mice required to detect significance with an alpha rate set at .05 in a standardly powered experiment. For imaging experiments, between 4 and 5 mice were imaged per experimental paradigm.
Data exclusions	No data was excluded from the analysis.
Replication	To increase replication of the C. elegans-based studies, we used large amount of worms (upto 100 worms/group) and had at least two repeats. For all other experiments that were carried out, all attempts at replication were successful.
Randomization	Animal/samples (mice) were assigned randomly to the various experimental groups, and mice were randomly selected for behavioral experiments.
Blinding	In data collection and analysis (e.g., worm behavioral studies, worm imaging data analysis, as well as imaging and data analysis of EM), the performer(s) was blinded with experimental design.

Reporting for specific materials, systems and methods

We require information from authors about some types of materials, experimental systems and methods used in many studies. Here, indicate whether each material, system or method listed is relevant to your study. If you are not sure if a list item applies to your research, read the appropriate section before selecting a response.

Materials & experimental systems

n/a	Involved in the study	
[ ]	[x]	Antibodies
[ ]	[x]	Eukaryotic cell lines
[x]	[ ]	Palaeontology
[ ]	[x]	Animals and other organisms
[x]	[ ]	Human research participants
[x]	[ ]	Clinical data

Methods

n/a	Involved in the study	
[x]	[ ]	ChIP-seq
[ ]	[x]	Flow cytometry
[x]	[ ]	MRI-based neuroimaging

Antibodies

Antibodies used	Antibodies used were: β-actin (Santa Cruz, #sc-1616), WRN (Santa Cruz, # sc-5629), CD38 (Santa Cruz, # sc-374650), CD157 (R&D systems, #AF4736), CD73 (R&D systems, #AF5795), PAR (TREVIGEN, #4336-BPC-100), PARP1 (Cell signaling, #9542), AMPK (Cell signaling, #5831), pAMPK (Thr172) (Cell signaling, #2535), pULK1 (Ser555) (Cell signaling, #5869), ULK1 (Cell signaling, #6439), p62 (Cell signaling, #39749), Bcl2L13 (ThermoFisher, # PA5-15043), LC3 (Novus, #NB100-2220), PSD95 (Cell signaling, #3450). All other antibodies were obtained from Cell signaling.
Validation	All the antibodies were validated for use in mouse/human tissues based on previous publications (Fang EF et a., Cell 2014; Fang EF et al., 2016; Iaccarino HF et al., Nature 2016; Lazarou M et al., Nature 2015; et al.,). Furthermore, detailed antibody validation profiles are available in the website of designated companies.

Eukaryotic cell lines

Policy information about cell lines

Cell line source(s)	The primary fibroblast cell lines HT01 (#AG09599) and the WS01 (#AG03141) cells were acquired from Coriell Institute. The WRN-KD cells were siRNA knockdown in HT01 cells using WRN human siRNA ologo duplex (CAT#: SR322215, Origene). Briefly, siRNAs were incubated in Optimem with 4 ml RNA Interferin (siRNA transfection reagent, Polyplus) per 1 ng RNA for 15 min and added to complete media for a final concentration of 30 nM siRNA. After 3-day incubation, cells were applied for further experiments. Knockdown efficiency was examined by western blot. All other primary human fibroblasts (detailed in Table S1)

	and blood samples were from Chiba University with ethical approval by the ethics committee of the Chiba University Graduate School of Medicine, and signed informed consent obtained from the patients.
Authentication	None of the cell lines used were authenticated.
Mycoplasma contamination	All cell lines tested negative for mycoplasma contamination
Commonly misidentified lines (See ICLAC register)	No commonly misidentified lines were used

Animals and other organisms

Policy information about studies involving animals; ARRIVE guidelines recommended for reporting animal research

Laboratory animals	Mice carrying Wrn-/- allele30 were maintained under standard laboratory conditions at the NIA with free access to water and standard diets. All mouse work was done in accordance with the guidelines and policies of the NIH NIA ACUC committee under our 361-LMG protocols, and we have complied with all relevant ethical regulations for animal testing and research. Also, WS C. elegans and fly models were used in this study.
Wild animals	No wild animals were used in this study.
Field-collected samples	No field-collected samples were used in this study.
Ethics oversight	All mouse work was done in accordance with the guidelines and policies of the NIH NIA ACUC committee under our 361-LMG protocols, and we have complied with all relevant ethical regulations for animal testing and research.

Note that full information on the approval of the study protocol must also be provided in the manuscript.

Flow Cytometry

Plots

Confirm that:

- [ ] The axis labels state the marker and fluorochrome used (e.g. CD4-FITC).
- [ ] The axis scales are clearly visible. Include numbers along axes only for bottom left plot of group (a 'group' is an analysis of identical markers).
- [ ] All plots are contour plots with outliers or pseudocolor plots.
- [x] A numerical value for number of cells or percentage (with statistics) is provided.

Methodology

Sample preparation	Materials and methods: For mitochondrial ROS, isolated neurons were stained with MitoSOX (3 µM for 30 min), followed by fluorescence determination using a flow cytometer (BD ACCURI C6 PLUS). Flow data were analyzed using FCS Express 4 software.
Instrument	Flow cytometer BD ACCURI C6 PLUS is used for data collection.
Software	Flow data were analyzed using FCS Express 4 software.
Cell population abundance	N/A
Gating strategy	see methods for details.

- [x] Tick this box to confirm that a figure exemplifying the gating strategy is provided in the Supplementary Information.
