# Supplementary figures and images for: NAD+ augmentation restores mitophagy and limits accelerated aging in Werner syndrome
Source: Nat Commun. 2019 Nov 21;10:5284. doi: 10.1038/s41467-019-13172-8 (PMC6872719; doi:10.1038/s41467-019-13172-8)

Fig. 2. i

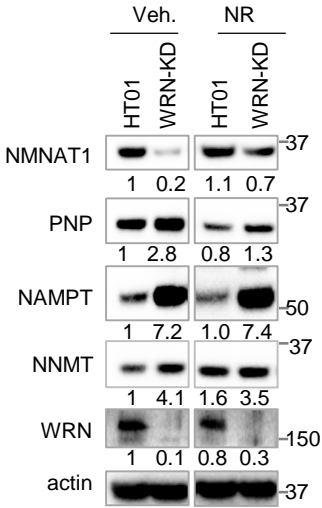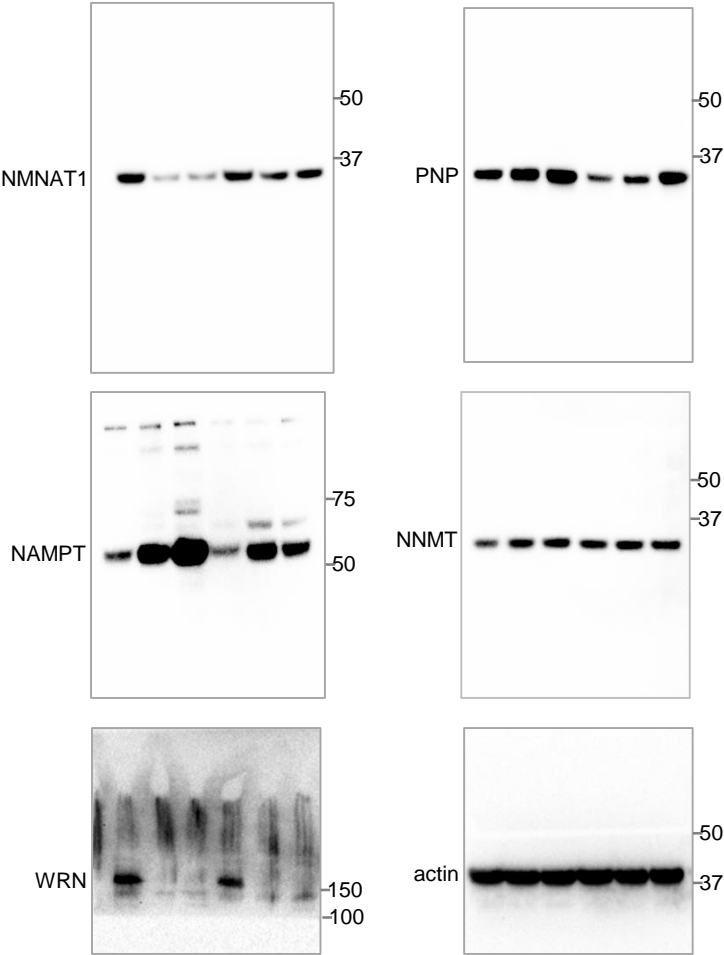

**Fig. 5. k**

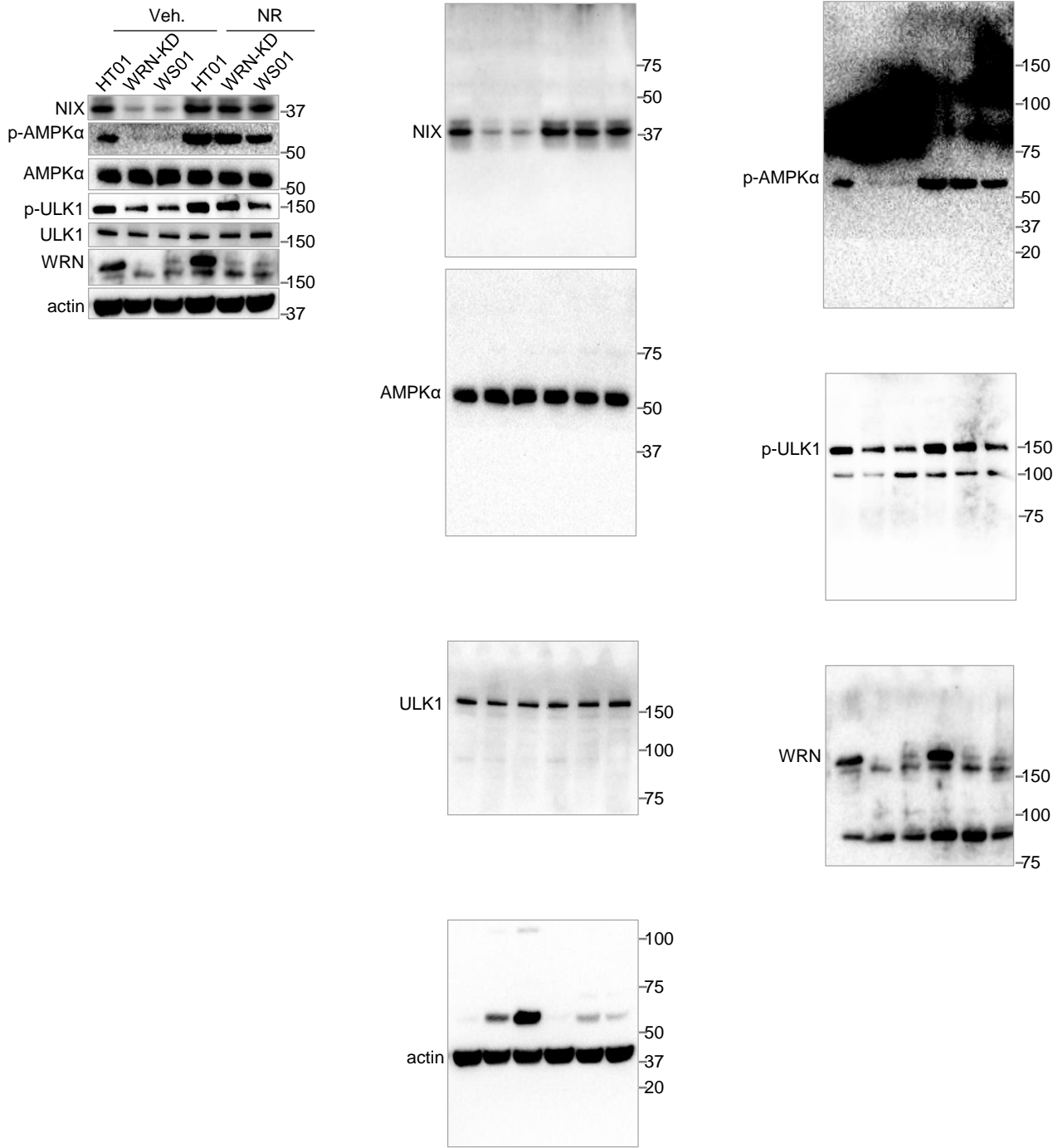

# Supplementary Figure 3k, l, m

**k**

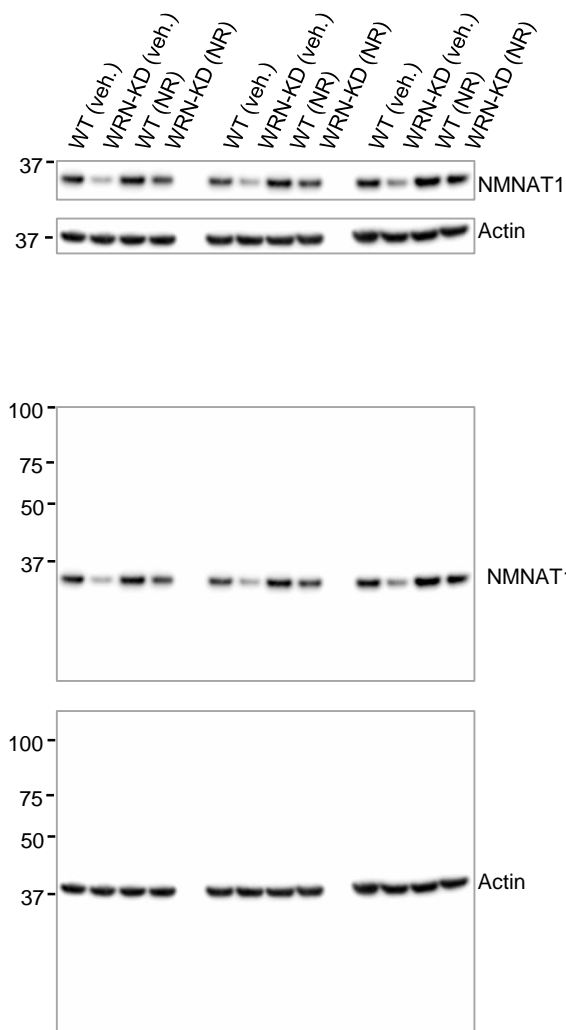

**l**

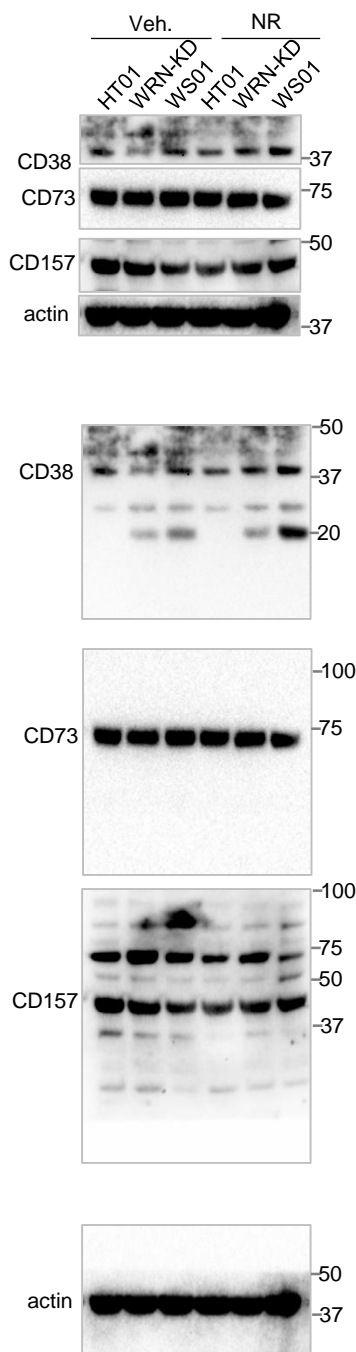

**m**

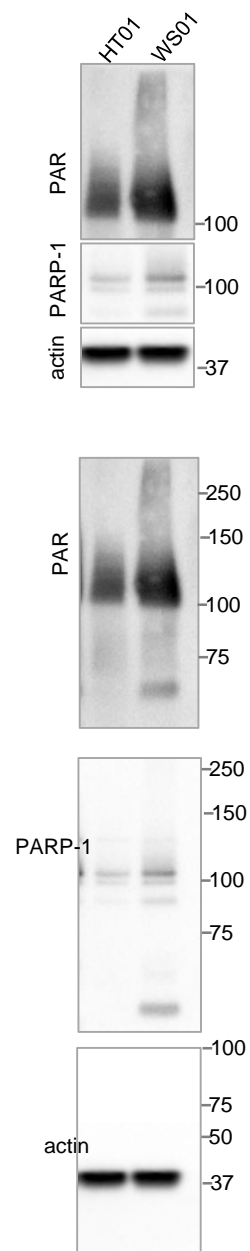

Supplementary Figure 8g

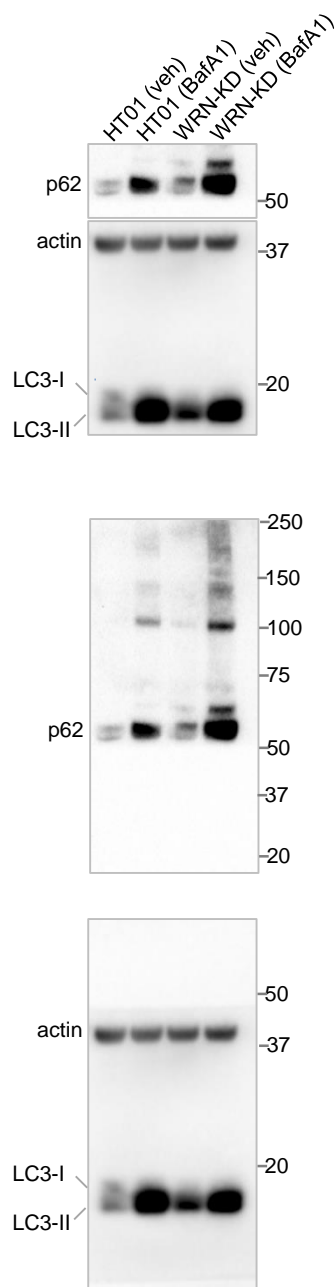

Supplement: Supplementary file 7 — Source Data [file 41467_2019_13172_MOESM7_ESM.zip › 183854_3_supp_4026269_pwnlb2.pdf]
